# In vitro interaction network of a synthetic gut bacterial community

**DOI:** 10.1038/s41396-021-01153-z

**Published:** 2021-12-02

**Authors:** Anna S. Weiss, Anna G. Burrichter, Abilash Chakravarthy Durai Raj, Alexandra von Strempel, Chen Meng, Karin Kleigrewe, Philipp C. Münch, Luis Rössler, Claudia Huber, Wolfgang Eisenreich, Lara M. Jochum, Stephanie Göing, Kirsten Jung, Chiara Lincetto, Johannes Hübner, Georgios Marinos, Johannes Zimmermann, Christoph Kaleta, Alvaro Sanchez, Bärbel Stecher

**Affiliations:** 1 Max von Pettenkofer Institute of Hygiene and Medical Microbiology, Faculty of Medicine, LMU, Munich, Germany; 2 Bavarian Center for Biomolecular Mass Spectrometry, TU Munich, Freising, Germany; 3 Department for Computational Biology of Infection Research, Helmholtz Center for Infection Research, Brunswick, Germany; 4 Department of Chemistry, Bavarian NMR Center–Structural Membrane Biochemistry, TU Munich, Garching, Germany; 5 Ramboll Deutschland GmbH, Munich, Germany; 6 Department of Microbiology, LMU, Martinsried, München, Germany; 7 Division of Paediatric Infectious Diseases, Dr. von Hauner Children’s Hospital, Ludwig Maximilians University, Munich, Germany; 8 Research Group Medical Systems Biology, Institute of Experimental Medicine, Christian-Albrechts-University Kiel, Michaelisstr. 5, 24105 Kiel, Germany; 9 Department of Ecology & Evolutionary Biology, Yale University, New Haven, CT USA; 10 Microbial Sciences Institute, Yale University, West Haven, CT USA; 11 German Center for Infection Research (DZIF), partner site LMU Munich, Munich, Germany

**Keywords:** Microbiome, Microbial ecology

## Abstract

A key challenge in microbiome research is to predict the functionality of microbial communities based on community membership and (meta)-genomic data. As central microbiota functions are determined by bacterial community networks, it is important to gain insight into the principles that govern bacteria-bacteria interactions. Here, we focused on the growth and metabolic interactions of the Oligo-Mouse-Microbiota (OMM^12^) synthetic bacterial community, which is increasingly used as a model system in gut microbiome research. Using a bottom-up approach, we uncovered the directionality of strain-strain interactions in mono- and pairwise co-culture experiments as well as in community batch culture. Metabolic network reconstruction in combination with metabolomics analysis of bacterial culture supernatants provided insights into the metabolic potential and activity of the individual community members. Thereby, we could show that the OMM^12^ interaction network is shaped by both exploitative and interference competition in vitro in nutrient-rich culture media and demonstrate how community structure can be shifted by changing the nutritional environment. In particular, *Enterococcus faecalis* KB1 was identified as an important driver of community composition by affecting the abundance of several other consortium members in vitro. As a result, this study gives fundamental insight into key drivers and mechanistic basis of the OMM^12^ interaction network in vitro, which serves as a knowledge base for future mechanistic in vivo studies.

## Introduction

The mammalian gastrointestinal tract harbors hundreds of bacterial species that occupy distinct ecological niches [1, 2]. Diversity and stable coexistence of community members after initial assembly result in the exclusion of invaders [3, 4]. Community assembly and stability are inherently driven by commensal or cooperative trophic interactions, in which metabolic by- or end products of one species are the resources for another one [5–7]. At the same time, bacteria compete for substrates by employing diverse predatory mechanisms, like the production of bacteriocins [8]. These interaction patterns form complex ecological networks and determine community-level functions of the microbiota including dietary breakdown, metabolite production, and colonization resistance [9–11]. Consequently, disruption of bacterial networks by antibiotics, disease, or diet-mediated interventions results in alterations of community-level functions [12, 13]. To be able to predict, preserve and manipulate microbial community function, it is important to identify functionally important members and understand relevant interaction mechanisms between individual bacteria.

A multitude of different approaches has been used to characterize the ecological networks of microbial communities. Function-related patterns in native microbial communities can be identified by systems biology approaches, combining metagenomics, metatranscriptomics, and metabolomics analyses [14]. Together with methods based on stable isotope probing, microorganisms with specific metabolic properties can be identified [15]. Potentially interacting species may be predicted from co-occurrence analysis supported by genome-guided metabolic modeling [16–18]. To experimentally verify the key ecological, structural, and functional role of certain species in driving community structure and function, synthetic microbial consortia provide several advantages over native communities. As they are well-characterized, scalable, and experimentally tractable, these systems are increasingly used to gain a mechanistic understanding of gut microbial ecology [19–22].

**Fig. 1:**
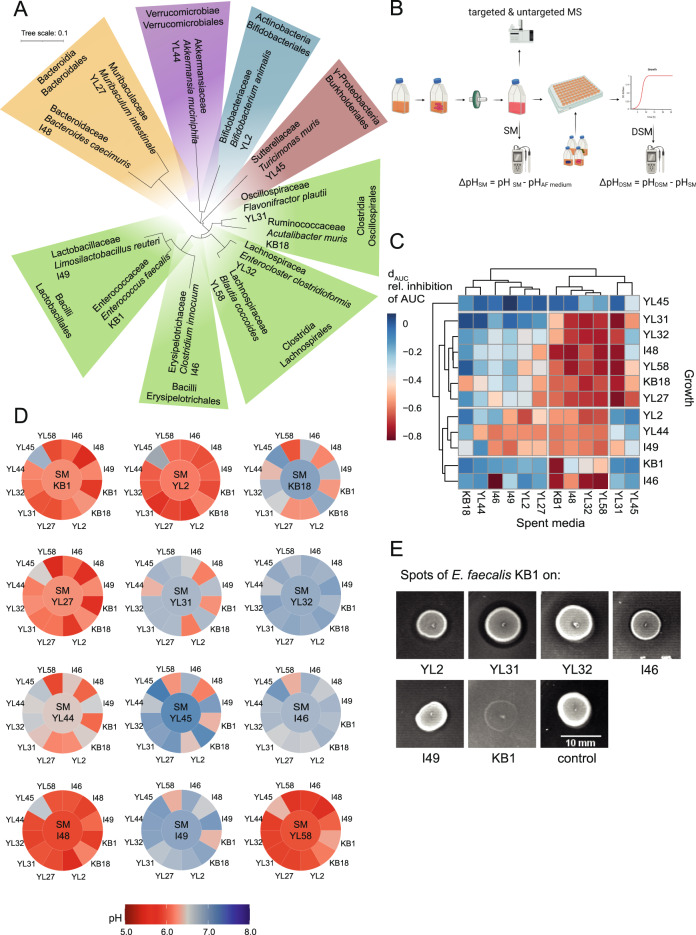
Growth analysis of OMM^12^ strains in spent media experiments. (**A**) Phylogenetic tree for bacteria of the OMM^12^ consortium based on the individual 16S rRNA genes. The consortium represents the five major phyla of the murine gastrointestinal tract: Firmicutes (green), Bacteroidetes (orange), Verrucomicrobia (purple), Actinobacteria (blue) and Proteobacteria (red). (**B**) Flowchart depicting spent culture medium (SM) preparation by growing bacterial monocultures in fresh AF medium for 20 h. Culture supernatants were sterile-filtered, samples for pH measurements and mass spectrometry were collected, and the SM was used as culture medium for the growth of all respective other strains. After growth of the individual strains in the specific SM, pH of the double spent medium (DSM) was determined. Differences in pH were then analyzed by calculating the corresponding ΔpH_SM_ and ΔpH_DSM_. (**C**) Monoculture growth in SM resulted in mostly decreased area under the growth curve (AUC) values in comparison to fresh AF medium, which was analyzed by calculating the inhibition factor d_AUC_. d_AUC_ was calculated from the mean AUC of three independent experiments relative to the mean AUC in fresh medium. (**D**) The mean pH of all SM (center) and DSM (outer tiles) after growth of the individual strains in fresh medium and the respective SM was determined from three independent experiments. Absolute values are available in SI data table 1. (**E**) Spot assays to test for production of antibacterial production. All bacterial strains of the OMM^12^ consortium were spotted onto a bacterial lawn of all the respective other strains. Inhibition zones were observed for *B. animalis* YL2, *F. plautii* YL31, *E. clostridioformi*s YL32, *C. innocuum* I46 and *L. reuteri* I49 when *E. faecalis* KB1 was spotted. No inhibition zone was seen for *E. faecalis* KB1 on itself. AF medium with *E. faecalis* KB1 spotted is shown as control.

The Oligo-Mouse-Microbiota (OMM^12^) is a synthetic bacterial community, which stably colonizes mice and provides colonization resistance against enteropathogen infection [23–26]. The OMM^12^ comprises twelve bacterial species (*Enterococcus faecalis* KB1, *Limosilactobacillus reuteri* I49, *Bifidobacterium animalis* YL2, *Clostridium innocuum* I46, *Blautia coccoides* YL58, *Enterocloster clostridioformis* YL32, *Flavonifractor plautii* YL31, *Acutalibacter muris* KB18, *Bacteroides caecimuris* I48, *Muribaculum intestinale* YL27, *Akkermansia muciniphila* YL44 and *Turicimonas muris* YL45), representing the five major eubacterial phyla in the murine gastrointestinal tract [27] (Fig. 1A). The model is openly available for non-commercial use [28], and is therefore increasingly employed in preclinical microbiome research [29–32]. So far, little is known about the system’s ecology and metabolic capabilities, both of which are factors that determine assembly, population dynamics, and bacterial community functionality. Therefore, we aimed for a comprehensive exploration of the metabolic potential (i.e., substrates, metabolism, and end products) and interactions between individual members of the OMM^12^ consortium. We employed a bottom-up approach connecting outcomes of mono- and pairwise co-culture experiments with observations from complex communities in in vitro batch culture. Furthermore, we combined metabolomics analysis of spent culture supernatants with genome-informed pathway reconstruction and generated draft metabolic models of the OMM^12^ consortium. Overall, we find that the majority of in vitro strain-strain interactions are amensalistic or competitive, which may be due to the environmental conditions in rich culture media. In accordance, bacteriocin production and substrate overlap between the individual strains was correlated with negative strain-strain interaction in vitro. Together, this work identified key interaction patterns among OMM^12^ strains relevant in community assembly and functionality.

## Results

### Probing directional interactions of OMM^12^ strains using spent culture media

To characterize directional interactions of the OMM^12^ consortium members, we chose an in vitro approach to explore how the bacterial strains alter their chemical environment by growth to late stationary phase.

Growth of the individual monocultures in a rich culture medium that supports growth of all members (AF medium, Methods, Table S1) was monitored over time (Fig. S1; SI data table 1) and growth rates (Table S2) were determined. Strains were grouped by growth rate (GR) into fast growing strains (GR > 1.5 h^–1^, *E. faecalis* KB1, *B. animalis* YL2, *C. innocuum* I46 and *B. coccoides* YL58), strains with intermediate growth rate (GR > 1 h^–1^, *M. intestinale* YL27, *F. plautii* YL31, *E. clostridioformis* YL32, *B. caecimuris* I48 and *L.reuteri* I49) and slow growing strains (GR < 1 h^–1^, *A. muris* KB18, *A. muciniphila* YL44 and *T. muris* YL45). All strains reached late stationary phase within 20 h of growth. To probe overlap in substrate requirements and interactions between the individual OMM^12^ members mediated by waste products or bacteriocins, sterile spent culture medium (SM) after growth to late stationary phase of all strains was obtained. Each OMM^12^ strain was cultured in the SM of the other community members and their own SM and growth rate, the area under the growth curve (AUC) and the pH were determined (Fig. 1B; Fig. S2; SI data table 1).

A normalized inhibition factor (d_AUC_) was determined by the AUC in SM relative to the AUC in fresh AF medium $( {{{{{{{{\mathrm{d}}}}}}}}_{{{{{{{{\mathrm{AUC}}}}}}}}} = \frac{{AUC_{SM} - AUC_{AF}}}{{AUC_{AF}}}})$ to quantify the influence of the different SM on the growth of the individual OMM^12^ strains (Fig. 1C). Ten of the twelve SM were found to enable decreased (d_AUC_ < –0.5) growth of at least one other strain of the consortium. Only the SM of strains *A. muris* KB18 and *A. muciniphila* YL44 enabled reduced growth of just the strains themselves. Corresponding to decreased AUC values in SM, growth rates were found to be lower as well, resulting in linear correlation of AUC and growth rates (Fig. S3, *R* > 0.5, *p* < 0.05 for all strains). The SM of four strains, *E. faecalis* KB1, *B. coccoides* YL58, *E. clostridioformis* YL32 and *B. caecimuris* I48, were found to strongly inhibit (d_AUC_ < –0.5) the growth of nine other strains each (Fig. 1C). Notably, growth of *E. faecalis* KB1 itself was only strongly reduced in its own SM, while it was able to grow in other strains’ SM. *T. muris* YL45 was the only strain not showing clear growth inhibition in any of the SM while its SM strongly decreased growth (d_AUC_ < –0.5) of three other strains, *A. muris* KB18, *M. intestinale* YL27 and *F. plautii* YL31.

### Individual pH profiles as indicators for niche modification

The pH of the culture medium after growth to stationary phase can be used as a measure for the extent of strain-specific environmental modification [11] and may partly explain inhibition of bacterial growth in a SM. Therefore, we determined the pH of the individual SM before and after (double spent media; DSM) growth of all OMM^12^ strains (Fig. 1B; SI data table 1). From these values, we defined the ΔpH for every strain after growth in fresh medium (ΔpH_SM_) and in all SM (ΔpH_DSM_) by analyzing the strength (difference of pH values) and direction (more acidic or more alkaline) of the pH change (Fig. 1D). After growth in fresh AF medium with neutral pH of 7.0, the OMM^12^ strains showed different degrees of ΔpH_SM_. While *E. faecalis* KB1, *B. animalis* YL2, *M. intestinale* YL27, *B. caecimuris* I48 and *B. coccoides* YL58 distinctly acidified the medium (pH_SM_ < 6.2), the growth of the other strains resulted in either slightly more alkaline or nearly neutral medium. Correlating inhibition of growth in a SM (d_AUC_) with the mean pH of the individual SM for each strain revealed that growth inhibition did not directly correlate with the pH. Only strains *B. animalis* YL2, *A. muciniphila* YL44 and *B. caecimuris* I48 showed a significant negative correlation (R < –0.5, *p* < 0.05) between growth inhibition and pH (Fig. S4; SI data table 1) with stronger inhibition in more acidic pH ranges. Testing monoculture growth in fresh AF medium with adjusted pH values from pH 5 to pH 7.5 revealed that these strains indeed show decreased growth rates and lower final OD values in medium with pH < 6.5 (Fig. S5; SI data table 1). pH sensitivity was further observed for *M. intestinale* YL27.

Most interestingly, many strains did not show the same magnitude or direction of alteration in pH when grown in SM of another strain (ΔpH_DSM_) compared to growth in fresh culture medium (ΔpH_SM_). This indicates an altered metabolic behavior of some strains in specific SM environments that differs from metabolic behavior in fresh AF medium (Fig. S6, Supplementary Text A).

### Production of antibacterial compounds by *E. faecalis* KB1

Growth inhibition in SM (Fig. 1C) may further be explained by the production of antimicrobial compounds. To test for the production of antimicrobial compounds by the OMM^12^ strains, we used a phenotyping approach and performed spot assays on agar plates (Fig. 1E). Inhibition zones were only seen in case of *E. faecalis* KB1, which produced one or several compounds active against *B. animalis* YL2, *F. plautii* YL31, *E. clostridioformis* YL32, *C. innocuum* I46 and *L. reuteri* I49. Genomic analysis revealed that the strain encodes genes for the production of several bacteriocins (Supplemental Text B), including enterocin L50, an enterococcal leaderless bacteriocin with broad target range among Gram-positive bacteria [33]. All other strain pairs did not show signs of growth inhibition by compound excretion under these conditions, despite the presence of genes for lanthibiotic production in the genome of *B. coccoides* YL58 (determined by antiSMASH) [34]. Although expression of antimicrobial molecules may be induced by specific environmental triggers, which are absent in the monoculture in vitro setting, we concluded that interference competition may only play a role in a subset of pair-wise interactions in AF medium involving *E. faecalis* KB1.

### Substrate depletion profiles correlate with growth inhibition in SM

**Fig. 2:**
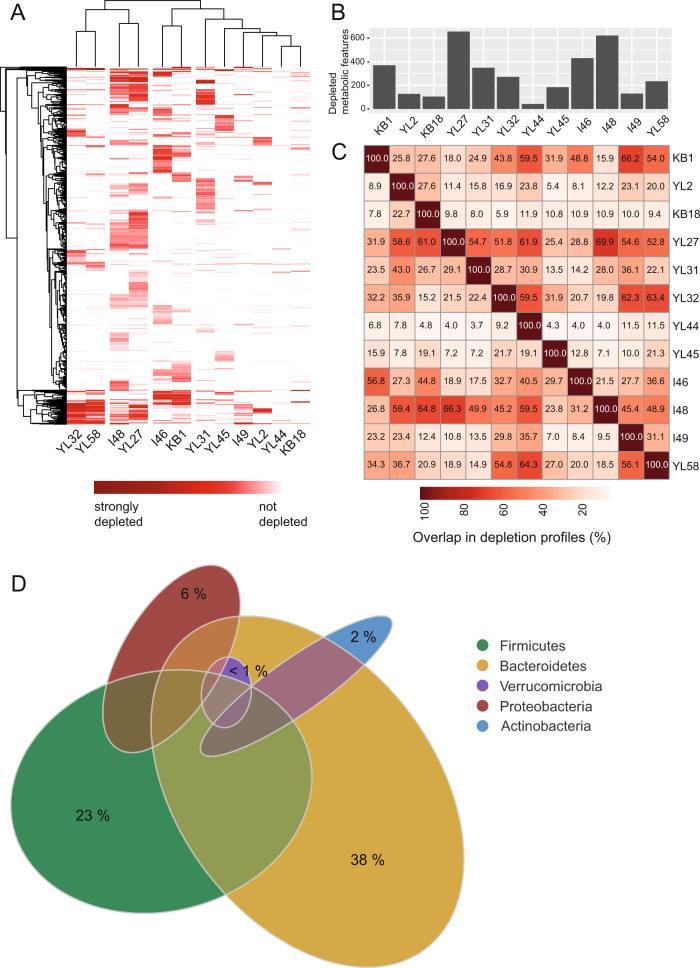
Overlap of substrate depletion profiles between individual OMM^12^ strains. (**A**) Depletion profiles of substrates after bacterial growth to stationary phase in AF medium were determined by untargeted MS from three independent experiments. All metabolomic features (rows) that significantly decreased (*p* < 0.05 compared to fresh media) compared to fresh medium for at least one of the twelve strains are shown in red. Dark-red indicates strong depletion, while white indicates no depletion of the metabolomics feature. Hierarchical clustering of strain-specific profiles as well as metabolomic features reveal profile similarities between phylogenetically similar strains. (**B)** Bar plot showing the total number of significantly (*p* < 0.05 compared to fresh media) depleted metabolomic features in AF medium for the individual strains. (**C**) Pairwise overlap in depleted metabolomic features relative to the total number of depleted metabolomic features (shown in **B**) of every individual strain. E.g., *E. faecalis* KB1 shares 33 metabolomic features from its set of 370 depleted metabolomic features with *B. animalis* YL2, corresponding to 8.9%. As *B. animalis* YL2 in contrast only depletes 128 metabolomic features in total from AF medium, this corresponds to an overlap of 25.8% of shared metabolites between *B. animalis* YL2 and *E. faecalis* KB1 relative to the total set of *B. animalis* YL2 depleted metabolomics features. (**D**) Euler diagram depicting number of depleted metabolomic features and overlap within the full consortium as grouped by bacterial phyla. Size of the ellipses denotes the number of depleted features, size of overlap between ellipses denotes number of features that are shared when comparing all individual profiles. Where several ellipses overlap, depleted metabolomic features are shared by more than two phyla. Colors indicated in the legend denote areas of metabolomics features that are unique to a phylum (indicated in percent of total depleted metabolomics features), overlapping areas are indicated in muted colors.

As pH and antimicrobial compounds only partly explained inhibition of growth in SM, we set out to gain more insights into the individual metabolic profiles in our in vitro setting. Therefore, triplicate samples of fresh AF medium and SM were analyzed by a mass spectrometry-based untargeted metabolomics approach (TripleTOF, Methods). Combining positive and negative ionization mode, 3092 metabolomic features were detected in total. From these, 2387 (77.20 %) were significantly altered (*t*-test, *p* value < 0.05) by at least one of the twelve strains (Fig. S7). Hierarchical clustering of the metabolomic feature depletion profiles (i.e., substrates used by the bacteria; Fig. 2A) reflects the phylogenetic relationship between the strains (Fig. 1A). Correlating the phylogenetic distance between the individual strains with the number of shared depleted metabolomic features in AF medium (Fig. S8) showed that phylogenetically similar strains of the consortium have a higher substrate overlap than phylogenetically distant strains (R = –0.29, *p* = 0.017). The total number of metabolomic features that are depleted from AF medium greatly varies for the different strains, ranging from over 600 depleted features for *M. intestinale* YL27 to only 42 for *A. muciniphila* YL44 (Fig. 2B). The number of metabolomic features overlapping with other OMM^12^ strains’ features relative to the strains’ total set of depleted features was determined (Fig. 2C). Phylogenetically related strains like *E. clostridioformis* YL32 and *B. coccoides* YL58 or *M. intestinale* YL27 and *B. caecimuris* I48 share over 50% of depleted metabolic features each, suggesting a strong substrate overlap in AF medium. Visualizing the extent of overlap between substrate depletion profiles reveals that Bacteroidales, Clostridia and Bacilli strains of the consortium dominate with the highest number of commonly depleted substrates in AF medium (Fig. 2D).

Correlating the growth inhibition in SM (d_AUC_) with the pairwise overlap in depletion profiles (Fig. 2C) revealed that a larger overlap is correlated with a stronger growth inhibition in the corresponding SM (R = –0.46, *p* = 3.1E–08, Fig. S9). This is illustrated by *A. muciniphila* YL44, which used only a low number of substrates from the AF medium (Fig. 2B) and the SM of which had only little effect on the growth of the other strains of the consortium (Fig. 1C). On the other hand, the strain´s growth itself was strongly reduced in the SM of most other consortium members (Fig. 1C, S2), which depleted a large spectrum of metabolomic features including those used by *A. muciniphila* YL44 (Fig. 2C).

### Genome-informed metabolic potential of the OMM^12^ consortium

**Fig. 3:**
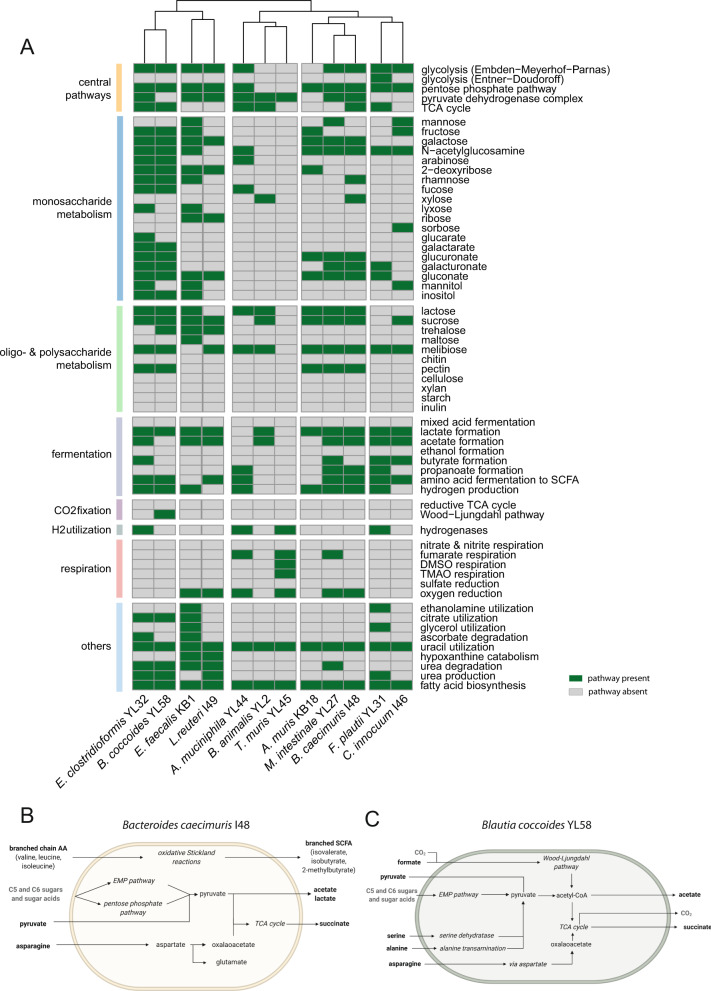
Metabolic potential of the OMM^12^ strains. **(**A) OMM^12^ metabolic models were reconstructed using gapseq [35] and gapseq output was screened for a hand-curated set of pathways to determine the strains’ potential to use a diverse range of substrate-specific and central pathways and release fermentation end products. Multiple pathways corresponding to the same function were grouped together according to the MetaCyc pathway database [36] (SI data table 2) and pathway utilization was considered positive (green) if one of the associated pathways was confirmed by gapseq. If none of the associated pathways were found, the potential substrate and pathway utilization was considered negative (grey). Metabolites and pathways were sorted by functional groups. By combining metabolomics data (MS, Fig. S10, S11) with genome-based information, broad-scale metabolic sketches of the individual OMM^12^ strains were generated (Fig. S12). Here, the models for strains *B. caecimuris* I48 (**B**) and *B. coccoides* YL58 (**C**) are shown exemplarily. Models of the remaining strains of the consortium are shown in Fig. S12. Experimentally confirmed substrates and products and pathways found by gapseq are shown in black. Hypothetical substrates, products, or pathways are shown in grey.

To gain insights into metabolic properties of the OMM^12^ strains, we reconstructed genome-scale metabolic models using gapseq [35] (SI data file). The initial metabolic models were curated by screening for metabolic pathways and transporter proteins and filling of missing reactions (gap-filling). From the genome-based metabolic models, we derived the presence and absence of metabolic pathways for central carbon metabolism (e.g., fermentation pathways, respiration), amino acid metabolism, and utilization of specific substrates, for the individual strains using MetaCyc pathways [36] (Fig. 3A, Fig. S10, SI data table 2). Further, the presence of specific substrate transporters was determined (Fig. S11, SI data table 2). Hierarchical clustering of the genome-informed metabolic potential (Fig. 3A) reflected their phylogenetic relationship in many instances. Generally, a high diversity of central and fermentation pathways was found among the consortium members. Moreover, enzymes for the degradation of amino acids (e.g., aspartate, glutamate, serine, and cysteine) are highly prevalent among consortium members. Correspondingly, systems for amino acid transport were especially prevalent among all strains of the consortium (Fig. S11).

### Metabolite production and fermentation pathways of the OMM^12^ strains in AF medium

To verify metabolites and fermentation products produced and consumed by the individual strains of the consortium in the given in vitro conditions, SM were analyzed using different mass spectrometry approaches (Fig. S12, S13). The combination of experimentally obtained insights and genome-based information on the presence of pathways was used to generate sketch drawings to visualize basic metabolic properties of the individual OMM^12^ community members (Fig. 3B, C, S14).

To confirm if fermentation pathways identified by genomics were active under in vitro conditions, short chain fatty acid (SCFA) production and consumption were analyzed (Fig. S12A). As observed for the SM metabolic profiles (Fig. S7), hierarchical clustering revealed that closely related bacteria showed similar SCFA production and consumption profiles. Both Bacteroidales strains produced acetic acid, succinic acid as well as branched-chain fatty acids. Both Lachnospiraceae strains generated high amounts of acetic acid. Butyric acid is produced by strains *F. plautii* YL31 and *C. innocuum* I46, the latter also being the only strain of the consortium excreting valeric acid and caproic acid. Of note, *F. plautii* YL31 also consumed lysine, indicating the ability to produce butyric acid from lysine, which was supported by the presence of the pathway for lysine fermentation to acetate and butyrate.

Formic acid was produced by several strains and consumed by *T. muris* YL45 and *B. coccoides* YL58. *B. coccoides* YL58 encodes genes for a CO dehydrogenase/acetyl-CoA-synthase, the key enzyme of the Wood-Ljungdahl pathway (reductive acetyl-CoA pathway). Formic acid can be processed via this pathway to acetyl-CoA. As another prominent example of bacterial fermentation, lactate production was confirmed (Fig. S12C) for *E. faecalis* KB1, *B. animalis* YL2, *F. plautii* YL31, *C. innocuum* I46, *E. clostridioformis* YL32 and *L. reuteri* I49, all of which harbor the pathway for lactate formation.

By quantifying amino acid levels we could show that Bacteroidales and Lachnospirales strains exhibited similar amino acid depletion and production profiles. In SM of strains *M. intestinale* YL27 and *B. caecimuris* I48, elevated levels of a diverse range of amino acids including glutamic acid, histidine, methionine, proline and phenylalanine were detected. Lachnospirales strains showed increased levels of isoleucine, tryptophan and valine, while alanine was especially depleted by *B. coccoides* YL58. Other strains of the consortium showed specific depletion of single amino acids, e.g., *F. plautii* YL31 strongly depleted lysine and glutamic acid, while *E. faecalis* KB1 depleted serine.

### Growth of OMM^12^ strains in pairwise co-culture

Next, we performed a set of experiments to characterize strain-strain interactions in the dynamic community-dependent context. We first analyzed direct competition of all strains in pair-wise co-cultures over the course of 72 h, with serial dilutions every 24 h. While growth was monitored continuously by OD 600 nm, samples for pH measurements and qPCR analysis were taken every 24 h. The growth curves of most co-cultures, as well as supernatant pH differed from the corresponding strain-specific characteristics observed in monoculture (Fig. S15, Fig. S16). These differences reflect co-culture dynamics, as can be seen from change in relative abundances over time.

**Fig. 4:**
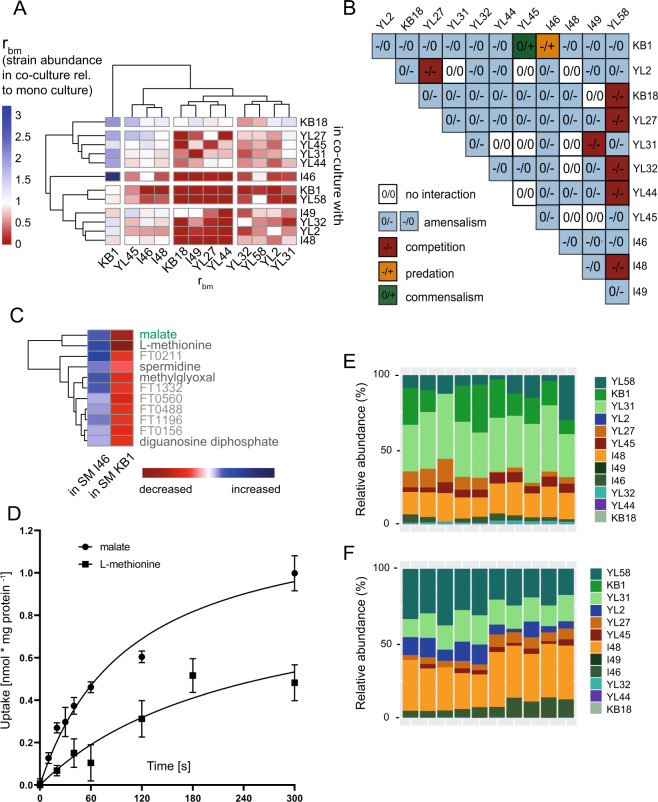
Transferring pairwise interactions to the community level. (**A**) OMM^12^ pairwise strain combinations (12 monocultures, 66 co-cultures) were cultured in a 1:1 ratio in fresh AF medium over the course of 72 h. Mean absolute abundance (normalized 16S rRNA gene copies determined by qPCR) after 72 h was determined. By comparing the mean bacterial abundance from three independent experiments in co-culture to the mean abundance in the corresponding monoculture, the factor r_bm_ was determined, as a measure of how successful a strain can grow in co-culture relative to monoculture after 72 h is shown. A ratio r_bm_ = 1 indicates no change in absolute abundance in the co-culture compared to mono culture. A ratio r_bm_ > 1 and a ratio r_bm_ < 1 indicate an increase and decrease in absolute abundance in the co-culture compared to mono culture, respectively. (**B**) Changes in the absolute abundance of a strain in co-culture compared to monoculture were determined and a pairwise interaction matrix was generated. Interactions where the individual abundance in co-culture significantly (*t*-test, *p* < 0.05) increased are indicated with (+), interactions where it significantly decreased are indicated with (–) and interaction where the abundance did not change in comparison to monoculture growth were indicated with (0). (**C**) Potentially cross-fed metabolites from *C. innocuum* I46 to *E. faecalis* KB1 were determined by comparing SM profiles (determined by untargeted MS) of KB1 and I46 for metabolites that are highly produced by I46 and consumed by KB1. Verified annotations are shown in green, potential annotations are shown in black and not annotated compounds are shown in grey as the corresponding feature identification numbers. (**D**) Time course of malate and L-methionine uptake by whole cells of *E. faecalis* KB1. Rates of ^14^C-malate uptake were measured at a final malate concentration of 10 µM at 18 °C. Standard deviations are shown from three biological replicates. (**E**) Using a serial passaging batch culture setup, the OMM^12^ community composition was analyzed after ten days of serial dilutions by comparing the relative strain abundances of ten replicates from two independent experiments in AF medium via qPCR. (**F**) Using the same approach, community composition of an OMM^11^-KB1 dropout community was analyzed after ten days of serial dilutions by comparing the relative strain abundances of ten replicates from two independent experiments in AF medium.

To identify directionality and mode of interaction between the OMM^12^ strains, we analyzed the relative changes in absolute abundance (normalized 16S rRNA gene copies) as a measure of how successful a strain can grow in co-culture relative to monoculture after 72 h. The mean absolute abundance ratio was calculated for every strain in all pairwise co-cultures $( {{{{{{{{\mathrm{r}}}}}}}}_{{{{{{{{\mathrm{i,bm}}}}}}}}}=\frac{{m_{i,co}(t72h)}}{{m_{i,mono}(t72h)}}} )$ (Fig. 4A, Methods). If absolute abundance of a strain increased significantly in co-culture relative to monoculture (r_bm_ > 1), the interaction was categorized as positive (+), if it decreased (r_bm_ < 1) the interaction was categorized as negative (–) (*t*-test comparing the r_bm_ of three independent experiments, Fig. S17). If it did not significantly (*p* > 0.05) differ from that in monoculture (r_bm_ = 1), the interaction was categorized as neutral (0). By this, we created a co-culture interaction matrix (Fig. 4B): the vast majority of the interactions was classified as amensalistic (0/– and –/0, 46 of 66 of interactions). A smaller subset of interactions was either competitive (–/–, 7 of 66 of interactions) or neutral (0/0, 11 of 66 of interactions). No mutualistic interactions (+/+) were observed. However, one example for each, commensalism (0/+ and +/0) and predation (+/– and –/+), were identified.

The extent to which the individual strains altered the growth of other community members in the co-culture differed distinctly. While *E. faecalis* KB1 and *C. innocuum* I46 lead to nine negative co-culture outcomes each, *A. muciniphila* YL44 and *A. muris* KB18 only impaired growth of one and zero strains, respectively. Simultaneously, both strains are negatively influenced in most co-cultures, with a significantly decreased absolute abundance in ten and eight co-cultures, respectively. Notably, *B. coccoides* YL58 is involved in five of seven competitive interactions of the consortium. These observations are in line with the outcomes observed in SM experiments, as strongly negative co-culture outcome corresponds to a strong inhibition of a strain in the respective SM (Fig. S18).

Of note, the only predatory interaction was observed between *C. innocuum* I46 to *E. faecalis* KB1, where the absolute abundance of *E. faecalis* KB1 significantly increased in the presence of *C. innocuum* I46 compared to monoculture growth. This beneficial interaction might be due to a metabolic advantage that arises in co-culture. In order to identify potentially cross-fed metabolites of *C. innocuum* I46 to *E. faecalis* KB1, we mined metabolomic data of SM for features enriched in *C. innocuum* I46 and depleted by *E. faecalis* KB1. Thereby, we identified several compounds including malate, L-methionine, spermidine, and methylglyoxal (Fig. 4C). To experimentally support the idea of cross-feeding, we exemplarily tested uptake of ^14^C-malate and ^3^H-L-methionine into intact cells of *E. faecalis* KB1 **(**Fig. 4D**)**. Both uptake of ^14^C-malate and uptake of ^3^H-L-methionine were strongly inhibited by the hydrophobic protonophores 2,4-dinitrophenol (DNP) and carbonyl cyanide m-chlorophenylhydrazone (CCCP), suggesting active transport driven by the proton motive force for both substrates. We found a very fast linear uptake of particularly ^14^C-malate within the first 60 s, which could explain why malate utilization confers a growth advantage to this strain. Decrease of absolute abundance of *C. innocuum* I46 in co-culture with *E. faecalis* KB1 might be due to the production of antimicrobial compounds by *E. faecalis* KB1 active against *C. innocuum* I46 (Fig. 1E).

### Community structure of the OMM^12^ consortium

Next, we set out to investigate if interactions found in co-cultures are transferrable to the strains’ behavior in the complete OMM^12^ community. To this end, all twelve OMM strains were simultaneously co-cultured in AF medium and serially diluted 1:100 every 24 h into fresh AF medium. Relative abundance of all strains after 10 days was determined by qPCR for ten replicates each in two independent experiments from different inocula (Fig. 4E).

While each of the OMM^12^ members except *E. faecalis* KB1 was outcompeted to a very low relative abundance in at least one pairwise culture (Fig. 4A, B), the majority (10 out of 12) of the consortium members were able to coexist in the complex community up to 10 days (Fig. 4E, Fig. S19A). Replicate communities showed reproducible community structure, even when different inocula were used. Interestingly, *F. plautii* YL31 dominated the community under these conditions. Furthermore, *B. coccoides* YL58 and *E. faecalis* KB1 showed a high relative abundance, which corresponds to their dominant role in SM and co-culture experiments (Figs. 1C, 4A, B). While strains *B. animalis* YL2 and *L. reuteri* I49 were not detectable at 10 days in all replicates, *A. muris* KB18 was found in only a few of the communities at 10 days (relative abundance < 1%).

### *E. faecalis* KB1 strongly impacts overall in vitro community composition

Following, we investigated how the absence of *E. faecalis* KB1, which plays a dominant role in pair-wise interactions, would affect the overall community structure. We generated a ''dropout'' community including all strains of the OMM^12^ consortium except *E. faecalis* KB1 (OMM^11^-*E. faecalis* KB1). Compositional analysis revealed increased relative abundance of *C. innocuum* I46 and *B. animalis* YL2 in the OMM^11^-*E. faecalis* KB1 compared to the full OMM^12^ community (Fig. 4F). In addition, the absolute abundances of strains *B. animalis* YL2, *C. innocuum* I46, *B. coccoides* YL58 and *B. caecimuris* I48 were found to increase significantly (*t-*test, *p* < 0.05) in the absence of *E. faecalis* KB1 (Fig. S19A (M1)). The increase in abundance of *B. animalis* YL2 and *C. innocuum* I46 may be explained by absent enterocin production or substrate competition by *E. faecalis* KB1. The latter may also explain increased abundance of *B. caecimuris* I48 and *B. coccoides* YL58 in the dropout community. Further, the abundance of *F. plautii* YL31, *E. clostridioformis* YL32, *A. muciniphila* YL44 and *T. muris* YL45 was found to decrease in the absence of *E. faecalis* KB1 (Fig. S19A (M1)). This indicates either direct positive effects of *E. faecalis* KB1 on these strains or indirect effects that occur through the overall shift in OMM^11^-*E. faecalis* KB1 community composition compared to the OMM^12^ consortium.

### Influence of specific supplements on community structure

**Fig. 5:**
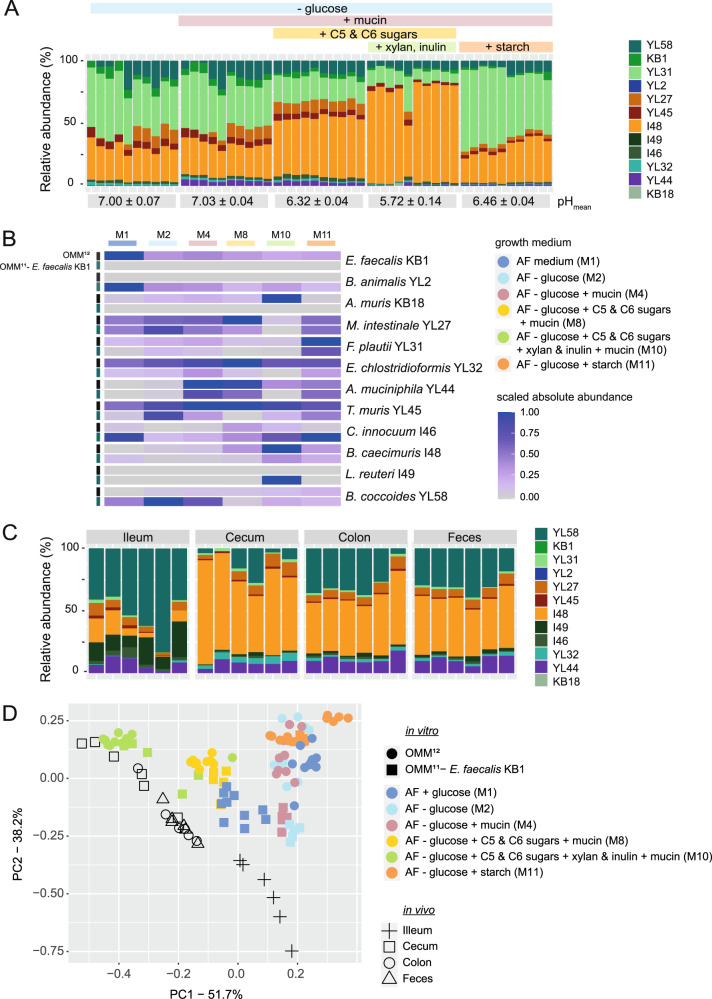
Influence of the nutritional environment on OMM^12^ community composition. (**A**) To study the influence of different media supplements on community composition, the OMM^12^ community composition was analyzed after ten days of serial dilutions in AF media with indicated supplements. The relative strain abundances of ten replicates from two independent experiments are shown. The mean pH of all culture supernatants at day ten is shown with the corresponding SD. (**B**) Absolute abundance of each strain in different media and inoculated communities (OMM^12^ and OMM^11^-*E. faecalis* KB1) were scaled for each individual strain to reveal trends in changes of abundance in the different experimental setups. Media conditions are shown in colors (**C**) OMM^12^ community composition in different gut regions of adult C57BL/6 mice. Mice were sacrificed at ZG 10 and content from different gut regions was processed for DNA extraction and qPCR. The relative strain abundances of 5 replicate mice in ileum, cecum, colon, and feces are shown. (**D**) PCA of community structure in different media and the mouse gut. Principle component analysis was performed on rel. abundance data of OMM^12^ and OMM^11^-*E. faecalis* KB1 community composition in different in vitro culture media and data of OMM^12^ community composition in vivo. Inoculated communities and gut regions are shown in different shapes, culture media compositions are shown in different colors.

Finally, we assessed the effect of media composition on community structure under our in vitro culture conditions. Therefore, we generated a comprehensive dataset comparing the composition of the complete OMM^12^ community and the *E. faecalis* KB1 dropout in media with different supplements that are known to promote the growth of specific gut bacteria but are missing in AF medium (mucin, C5/C6 sugars, xylan & inulin, starch; Figs. 5A, B, S19). We used a modified AF medium with reduced glucose concentration to rule out that substrate consumption may be inhibited by catabolite repression. Of note, reduction of glucose resulted in decrease of *E. faecalis* KB1, *C. innocuum* I46, and *B. animalis* YL2 and increase of *A. muciniphila* YL44 (Figs. 5B, S19). We found that the chosen supplements had characteristic effects on relative and absolute abundance of individual strains (Figs. 5A, B, S19). *A. muciniphila* YL44, a known mucin-degrader, was boosted by mucin. Additional supplementation with C5/C6 sugars (xylose, arabinose, lyxose, fucose and rhamnose) promoted growth of *B. caecimuris* I48. Further, supplementing media with xylan and inulin promoted growth of *A. muris* KB18 and even more enhanced levels of *B. caecimuris* I48. At the same time, *A. muciniphila* YL44 and *M. intestinale* YL27 were decreased. Interestingly, *L. reuteri* I49 was also promoted by xylan & inulin but only in the OMM^11^-*E. faecalis* KB1 dropout community (Figs. 5B, S19). Finally, supplementation of AF medium with starch only promoted growth of *F. plautii* YL31 (Figs. 5A, B; S19). Of note, increase of *C. innocuum* I46 and *B. animalis* YL2 strains was generally observed in the *E. faecalis* KB1 dropout communities irrespective of the media conditions. Lack of *E. faecalis* KB1 had different effects on the abundance of *B. caecimuris* I48, *M. intestinale* YL27 and *F. plautii* YL31 depending on supplements (Fig. 5A, B, S19).

### Comparison of in vitro and in vivo OMM^12^ community structure

In order to evaluate, which of the in vitro conditions most closely resemble community composition in the murine gut, we analyzed community composition in the ileum, cecum, colon, and feces in 5 age-matched adult (12 weeks old) male C57Bl/6 mice. In general, intestinal communities were similar in cecum, colon, and feces, with *B. caecimuris* I48, *B. coccoides* YL58 and *A. muciniphila* YL44 predominating (Fig. 5C). The ileal community was distinct and dominated by *B. coccoides* YL58 and *L. reuteri* I49. The relative abundance of *E. faecalis* KB1 and *F. plautii* YL31 was comparatively low in in vivo samples. Similar as in AF media, *A. muris* KB18 and *B. animalis* YL2 were at the detection limit of the qPCR assay. Principal Component Analysis (PCA) of relative abundance data was used to compare in vivo community structures to OMM^12^ and OMM^11^-*E. faecalis* KB1 cultured in vitro in different media (Fig. 5D). The highest similarity was observed between OMM^12^ community structure in cecum content and AF medium supplemented with mucin, C5/C6 sugars, xylan, and inulin. Conversely, OMM^12^ community structure in feces and colonic content most closely resembled OMM^12^ and/or OMM^11^-KB1 in medium M1 and M10. Taken together, we identified specific supplements that can be used to shape in vitro conditions to more closely recapitulate community structure in different regions of the murine gut.

## Discussion

A central challenge in gut microbiome research is to understand how interactions between the individual microorganisms affect community-level structure and related functions. Bottom-up approaches involving synthetic communities are valuable tools to study these interactions, as they allow to reduce complexity and to enable strain-specific manipulation. Using an in vitro approach, we focused on characteristics and interactions of the OMM^12^ community and combined monoculture, pairwise, and community cultivation of the strains with genome and metabolomics analysis of their spent media. Thereby we reveal that the OMM^12^ community interaction network is shaped by exploitative and interference competition in nutrient-rich culture media. In particular, *E. faecalis* KB1, a low-abundant member of the mammalian gut microbiota, was identified as an important driver of in vitro community composition by directly or indirectly altering the abundance of several other consortium members. We demonstrate that in vitro community structure can be modulated by specific supplements to more closely resemble the community in the murine gut. Additionally, we provide metabolic network reconstructions of the individual OMM^12^ strains, which are readily usable for *in silico* simulations and mechanistic studies using this synthetic community.

Exploitative (i.e., substrate) competition plays a major role in shaping intestinal bacterial communities [37]. Understanding the underlying principles of how bacteria compete for available nutrients is essential to predict and control community composition. We found that phylogenetically similar strains showed a higher substrate overlap (Fig. S8), which is in accordance with previous studies demonstrating that phylogeny reflects the metabolic capabilities of bacteria [38, 39]. Furthermore, overlap in substrate depletion profiles was correlated with growth inhibition in the respective SM (Fig. S9). This clearly indicates strong exploitative competition between individual OMM^12^ strains under the chosen in vitro conditions. We would like to note that the choice of culture media is an important deterministic factor of the observed competitive interactions and nutrient limitation may lead to more mutualistic interactions. *B. caecimuris* I48, *E. faecalis* KB1, *E. clostridioformis* YL32 and *B. coccoides* YL58 were found to consume a high number of substrates (>200), while their SM inhibited growth of the majority of the other community members including themselves (Fig. 1C). Of note, *M. intestinale* YL27 and *C. innocuum* I46 also consumed over 200 substrates each, but inhibited a few other strains. This demonstrates that substrate overlap cannot simply predict inhibition in all cases and other mechanisms (e.g., waste product inhibition) play a role in specific cases.

Besides substrate competition, a strain’s ability to acidify its environment or release an inhibitory factor (e.g., waste products, bacteriocins) can determine if another species can grow in the exhausted medium or not. Several strains, including *B. caecimuris* I48, *B. coccoides* YL58, *E. faecalis* KB1 and *C. inocuum* I46 acidified the medium during growth in monoculture. However, only for a few species, *A. muciniphila* YL44, *B. caecimuris* I48 and *B. animalis* YL2, acidic pH correlated with reduced growth (Fig. S4). Indeed, as reported previously, growth rate of these bacteria was strongly reduced at acidic pH [40] (Fig. S5). Moreover, the pH in the full OMM^12^ community in glucose rich AF medium, where most of the strains coexisted, was also slightly acidic (pH of 6.2), suggesting that pH modification does not play a major role in driving community composition under the conditions chosen. Interference competition by bacteriocins is widespread among gut bacterial communities [41]. We found that *E. faecalis* KB1 produces at least one antimicrobial compound that shows activity against five of the Gram-positive OMM^12^ strains (*B. animalis* YL2, *E. clostridioformis* YL32, *F. plautii* YL31, *C. innocuum* I46 and *L. reuteri* I49) (Fig. 1E). *E. faecalis* harbors genes coding for at least two different enterocins (enterocin L50A/L50B and enterocin O16). Therefore, we hypothesize that some of the inhibitory effects of *E. faecalis* KB1 on those strains can be attributed to enterocin-mediated killing. Generation of targeted deletions of the respective genes in *E. faecalis* will be required to prove that this mechanism indeed plays a role.

*E. faecalis* is a prevalent but low-abundant member of the undisturbed human and animal microbiota. Following antibiotic therapy, the bacterium can dominate the gut and cause blood-stream infection in immunocompromised individuals [42]. Understanding how *E. faecalis* out-competes/overgrows other gut microorganisms is important in order to intervene with *E. faecalis* domination in the gut. Besides antimicrobial-mediated inhibition of other bacteria our data suggest that metabolite cross-feeding contributes to the interaction of *E. faecalis* KB1 with *C. innocuum* I46 (Fig. 4A, B; Fig. S17). Based on metabolic profile mining we hypothesize that *E. faecalis* KB1 consumes malate, methionine, arginine and serine among other metabolites in co-culture with *C. innocuum* I46 (Fig. 4C). These metabolites may be secreted by *C. innocuum* I46 or alternatively, become available upon antimicrobial-mediated lysis. Interestingly, a previous study [43] reported that glucose-malate co-metabolism increases the growth of *E. faecalis* over glucose consumption alone. In connection with fast uptake rate of ^14^C-malate by *E. faecalis* KB1 (Fig. 4D), this suggests that malate cross-feeding may also contribute to *E. faecalis* KB1 gain in absolute abundance in co-culture with *C. inoccuum* I46. In addition, *E. faecalis* KB1 may benefit from increased availability of shared substrates that become available upon enterocin-dependent inhibition. These mechanisms will have to be dissected in future work using *E. faecalis* KB1 mutants in enterocin production and metabolite uptake systems.

While dominating the in vitro community in glucose-rich complex media, the relative abundance of *E. faecalis* KB1 in different gut regions of adult mice was very low (<1%; Fig. 5). This raises the question whether in vitro observations can be translated to its role in the microbiota of adult mice. Encouraged by published work that reports high relative abundance of *E. faecalis* in newborn mice [44], we analyzed community composition in 7-day old OMM^12^ mice (Fig. S20). Of note, *L. reuteri* I49 and *E. faecalis* KB1 dominated the small intestinal community in these mice, suggesting that the environmental conditions in the neonatal gut foster growth of these *Lactobacillales* species. Most other OMM strains including *B. animalis* YL2, which is usually found in infant mice, were below the detection limit of our method. We conclude that the neonatal gut could be a highly relevant environment to study *E. faecalis* KB1 ecology due to its high abundance.

Using batch culture experiments we were able to investigate the assembly and dynamics of full OMM^12^ and OMM^11^-*E. faecalis* KB1 dropout communities in vitro. A significant increase in *B. animalis* YL2 and *C. innocuum* I46 in the community lacking *E. faecalis* KB1 suggested that enterocin-mediated killing and/or substrate competition could also shape the more complex community. Of note, the effect was absent in medium M8 containing additional C5/C6 sugars (Fig. S19). This suggests that both strains can co-exist when they do not have to compete for the same limiting substrate. Notably, in the full OMM^12^ community, ten of the twelve strains co-existed over ten days. This was remarkable given the high number of negative pairwise interactions in SM and co-culture experiments. Differences between the behavior of strains in pairwise versus complex communities point at higher-order ecological interactions that emerge in the community context. As previously shown in other studies, the underlying mechanisms may involve metabolic flexibility or mixed substrate utilization of the strains in the presence of competitors [45], metabolite cross-feeding, lack of waste-product inhibition, and overall change in pH [11] or an excess of provided substrates in the medium.

Following up, it will be important to assess if the in vitro findings can be translated to the mouse model. The choice of culture media that mimic in vivo substrate range and physiology is clearly a critical factor. It is well known that the environmental conditions vary along the length of the gastrointestinal tract. Accordingly, we also observed different OMM^12^ community in the ileum, cecum, colon and feces (Fig. 5C, D). For in vitro characterization, we have deliberately chosen AF media (M1), because it supports growth of all 12 OMM strains (Fig. S1) and similar media have also been used in previous studies ([46, 47]). Mucin, which is known to support growth of *A. muciniphila* and others [48, 49], has been explicitly omitted due to its side effects. First, it strongly increases culture turbidity which interferes with growth measurements. Moreover, commercially available mucin contains dead bacteria, which impedes routine sterility tests by microscopy and PCR. However, as addition of mucin to culture media clearly increases abundance of *A. muciniphila* YL44 in batch cultures, we recommend using it for future mechanistic in vitro studies of this model. Moreover, addition of C5/C6 sugars may also be considered as it boosts growth of *B. cecimuris* I48 and shapes in vitro community structure to more closely mimic structure in the murine cecum. Of note, *F. plautii* YL31, which shows low abundance in vivo, was strongly promoted by the addition of starch to the media (Fig. 5A), despite the lack of known pathways for starch utilization (Fig. 3A). We also found examples of functions predicted by the metabolic models (e.g., SCFA production) that could not be confirmed experimentally (Fig. 3A). This clearly shows that future work is needed to refine metabolic models with help of experimental data and identify and characterize hitherto unknown metabolic pathways. Apart from that, we have to study regulation of metabolic pathways under changing environmental in order to better predict the impact of environmental conditions on microbial community structure.

Concluding, our work presents a comprehensive in vitro investigation of strain-strain interactions between members of a widely used synthetic intestinal bacterial community. Characterization of the metabolic profile of individual strains of the consortium as well as analyzing their metabolism and community assembly in co-culture revealed *E. faecalis* KB1 and *B. coccoides* YL58 to be important drivers of community composition. Drawing on this detailed understanding of in vitro behavior, our results will enable to employ this model for mechanistic in vivo studies. This step-wise approach may ultimately allow accurate description of interaction dynamics of in vivo gut microbial communities and pave the way for targeted manipulation of the microbiome to promote human health. In particular, extending the approach of dropout communities lacking specific strains could help to elucidate the role of individual players in community functions like dietary breakdown, metabolite production and colonization resistance and to identify general principles of how bacterial interaction networks and the corresponding emergence of higher order interactions shape microbiome function. This will enable the design of therapeutic interventions to control microbial community functions by advanced microbiome engineering.

## Methods

### Generation of a 16S rRNA gene-based phylogenetic tree

The genomes of the twelve strains of the OMM^12^ consortium [27] were accessed via DDBJ/ENA/GenBank using the following accession numbers: CP022712.1, NHMR02000001-NHMR02000002, CP021422.1, CP021421.1, NHMQ01000001-NHMQ01000005, NHTR01000001-NHTR01000016, CP021420.1, NHMP01000001-NHMP01000020, CP022722.1, NHMU01000001-NHMU01000019, NHMT01000001-NHMT01000003, CP022713.1 and annotated using Prokka (default settings) [50]. The 16S rRNA sequences of all strains were obtained. These rRNA FASTA sequences were uploaded to the SINA Aligner v1.2.11 [51] to align these sequences with a minimum 95% identity against the SILVA database. By this, a phylogenetic tree based on RAxML [52], GTR Model and Gamma rate model for likelihood was reconstructed. Sequences with less than 90% identity were rejected. The obtained tree was rooted using *midpoint.root()* in the phytools package [53] in R and visualized using iTOL online [54].

### Generation of genome-based metabolic models

Metabolic models were reconstructed using gapseq (1.1) with default settings [35]. In more detail, pathways were predicted as defined by the MetaCyc pathway database [36] and transporter were predicted based on the Transporter Classification Database (TCDB)[55] by using a bitscore cutoff of 200 for homology search. In agreement with experimental findings, the metabolic models were gap filled to allow in silico growth on AF medium which was prepared for computational applications accordingly [56]. The specific medium composition can be found in the supplementary file (AAM_co.csv) and is encoded in the provided SBML models.

The individual components of the medium—and their respective quantities—were estimated based on the literature and assigned to the respective exchange reactions of the models. Shortly, the brain-heart infusion was inferred based on the composition of meat extract [57, 58]; the constituents of the trypticase soy broth can be found in the TSB medium in the gapseq repository [35]; the components of the yeast extract is published along with the above-mentioned protocol [56], and the carbohydrate and amino acid composition of fetal calf serum originates from the amino acid composition of selected Bos taurus proteins (NCBI Reference Sequence, https://www.ncbi.nlm.nih.gov/) along with textbook information [59]. Mucins were represented based on the respective mucus composition as done previously [60].

A list of all MetaCyc pathways and their occurrence in the OMM^12^ strains was pulled from the gapseq models and filtered to remove all pathways with an expected taxonomic range of eukaryotes only. Multiple pathways performing the same function were grouped according to the MetaCyc classification hierarchy, excluding compounded superpathways. The pathway groups shown in Figs. 3 and S10 as well as the transporters in Fig. S11 were chosen manually according to their interest for the manuscript and referring to the metabolic data.

### Strains and culture conditions

Bacterial cultures were prepared from frozen monoculture stocks in a 10 ml culture and subculture in cell culture flasks (flask T25, Sarstedt) previous to all experiments. Cultures were incubated at 37 °C without shaking under strictly anaerobic conditions (gas atmosphere 7% H_2_, 10% CO_2_, 83% N_2_). All experiments, except experiments testing the influence of specific substrates on community composition, were carried out using AF medium (18 g.l^–1^ brain-heart infusion (Oxoid), 15 g.l^–1^ trypticase soy broth (Oxoid), 5 g.l^–1^ yeast extract, 2.5 g.l^–1^ K_2_HPO_4_, 1 mg.l^–1^ haemin, 0.5 g.l^–1^ D-glucose, 0.5 mg.l^–1^ menadione, 3% heat-inactivated fetal calf serum, 0.25 g.l^–1^ cysteine- HCl‧H_2_O). For experiments testing the influence of specific substrates on community composition, AF medium was prepared with 15 g.l^–1^ trypticase soy broth without added glucose (USBiological), as well as with the following substitute for brain-heart infusion: 6 g.l^–1^ meat extract, 8 g.l^–1^ nutrient broth (Difco) and 1.25 g.l^–1^ Na_2_HPO4. Using an enzyme-based colorimetric assay (Invitrogen, EIAGLUC) glucose concentration in this glucose-reduced AF medium was determined as 0.6 g/L. Depending on the experimental condition mucin was added in a final concentration of 0.025%, fucose, xylose, arabinose, rhamnose and lyxose were added 0.5 g/L each, xylan from beechwood (Roth) and inulin from chicory extract (Roth) were added 2 g/L each and starch (Roth) was added 2 g/L.

For pH adjustment (Fig. S5), media was titrated with diluted NaOH and HCl.

The following strains were used in this study: *Enterococcus faecalis* KB1 (DSM32036), *Bifidobacterium animalis* YL2 (DSM26074), *Acutalibacter muris* KB18 (DSM26090), *Muribaculum intestinale* YL27 (DSM28989), *Flavonifractor plautii* YL31 (DSM26117), *Enterocloster clostridioformis* YL32 (DSM26114), *Akkermansia muciniphila* YL44 (DSM26127), *Turicimonas muris* YL45 (DSM26109), *Clostridium innocuum* I46 (DSM26113), *Bacteroides caecimuris* I48 (DSM26085), *Limosilactobacillus reuteri* I49 (DSM32035), *Blautia coccoides* YL58 (DSM26115).

### Growth measurements

Bacterial growth was measured in 96well round bottom plates (Nunc) using a GenTech Epoch2 plate reader. Inocula were prepared from a previous culture and subculture and diluted in fresh AF medium to 0.01 OD_600nn_. Absorption at wavelength 600 nm was determined in a reaction volume of 100 μl in monoculture and SM experiments and 150 μl in co-culture experiments. During continuous measurements, the plate was heated inside the reader to 37 °C and a 30 s double orbital shaking step was performed prior to every measurement.

### Generation of spent culture media

Bacterial cultures and subcultures were grown for 24 h each in 10 ml AF medium at 37 °C under anaerobic conditions without shaking. Bacterial spent culture supernatants (SM) were generated by centrifugation of the densely grown subculture at 4 °C for 20 min at 5000 x *g* and subsequent pH measurement and filter-sterilization (0.22 μm). SM samples were aliquoted and immediately frozen at –80 °C. Samples were thawed under anaerobic conditions previous to growth measurements. Growth of all bacterial monocultures in the spent culture media (SM) of all respective other strains was then measured as described above. SM were inoculated with bacterial monocultures with starting OD_600nm_ 0.01. After monoculture growth of 20 h in the respective SM (resulting in double-spent media, DSM), pH values were determined.

### pH measurements

pH measurements of bacterial supernatants were performed using a refillable, glass double junction electrode (Orion™ PerpHecT™ ROSS™, Thermo Scientific).

### Metabolic profiling of late stationary phase bacterial supernatants

The untargeted analysis was performed using a Nexera UHPLC system (Shimadzu) coupled to a Q-TOF mass spectrometer (TripleTOF 6600, AB Sciex). Separation of the spent media was performed using a UPLC BEH Amide 2.1 × 100, 1.7 µm analytic column (Waters Corp.) with 400 µL/min flow rate. The mobile phase was 5 mM ammonium acetate in water (eluent A) and 5 mM ammonium acetate in acetonitrile/water (95/5, v/v) (eluent B). The gradient profile was 100% B from 0 to 1.5 min, 60% B at 8 min and 20% B at 10 min to 11.5 min and 100% B at 12 to 15 min. A volume of 5 µL per sample was injected. The autosampler was cooled to 10 °C and the column oven heated to 40 °C. Every tenth run a quality control (QC) sample which was pooled from all samples was injected. The spent media samples were measured in a randomized order. The samples have been measured in Information Dependent Acquisition (IDA) mode. MS settings in the positive mode were as follows: Gas 1 55, Gas 2 65, Curtain gas 35, Temperature 500 °C, Ion Spray Voltage 5500, declustering potential 80. The mass range of the TOF MS and MS/MS scans were 50–2000 m/z and the collision energy was ramped from 15–55 V. MS settings in the negative mode were as follows: Gas 1 55, Gas 2 65, Cur 35, Temperature 500 °C, Ion Spray Voltage –4500, declustering potential –80. The mass range of the TOF MS and MS/MS scans were 50–2000 m/z and the collision energy was ramped from –15–55 V.

The “msconvert” from ProteoWizard [61] were used to convert raw files to mzXML (de-noised by centroid peaks). The bioconductor/R package xcms [62] was used for data processing and feature identification. More specifically, the matched filter algorithm was used to identify peaks (full width at half maximum set to 7.5 s). Then the peaks were grouped into features using the “peak density” method [62]. The area under the peaks was integrated to represent the abundance of features. The retention time was adjusted based on the peak groups presented in most of the samples. To annotate possible metabolites to identified features, the exact mass and MS2 fragmentation pattern of the measured features were compared to the records in HMBD [63] and the public MS/MS database in MSDIAL [64], referred to as MS1 and MS2 annotation, respectively. The QC samples were used to control and remove the potential batch effect, *t*-test was used to compare the features’ intensity from spent media with fresh media.

The associated untargeted metabolomics data are available on MetaboLights repository [65] with ID MTBLS3535.

### Targeted short chain fatty acid (SCFA) measurement

The 3-NPH method was used for the quantitation of SCFAs [66]. Briefly, 40 µL of the SM and 15 µL of isotopically labeled standards (ca 50 µM) were mixed with 20 µL 120 mM EDC HCl-6% pyridine-solution and 20 µL of 200 mM 3-NPH HCL solution. After 30 min at 40 °C and shaking at 1000 rpm using an Eppendorf Thermomix (Eppendorf, Hamburg, Germany), 900 µL acetonitrile/water (50/50, v/v) was added. After centrifugation at 13,000 U/min for 2 min the clear supernatant was used for analysis. The same system as described above was used. The electrospray voltage was set to –4500 V, curtain gas to 35 psi, ion source gas 1 to 55, ion source gas 2 to 65, and the temperature to 500 °C. The MRM-parameters were optimized using commercially available standards for the SCFAs. The chromatographic separation was performed on a 100 × 2.1 mm, 100 Å, 1.7 μm, Kinetex C18 column (Phenomenex, Aschaffenburg, Germany) column with 0.1% formic acid (eluent A) and 0.1% formic acid in acetonitrile (eluent B) as elution solvents. An injection volume of 1 µL and a flow rate of 0.4 mL/min was used. The gradient elution started at 23% B which was held for 3 min, afterward the concentration was increased to 30% B at 4 min, with another increase to 40%B at 6.5 min, at 7 min 100% B was used which was held for 1 min, at 8.5 min the column was equilibrated at starting conditions. The column oven was set to 40 °C and the autosampler to 15 °C. Data acquisition and instrumental control were performed with Analyst 1.7 software (Sciex, Darmstadt, Germany).

### Dynamic metabolic profiling of bacterial supernatants

All chemicals were purchased from Sigma Aldrich at the highest purity available. 50 µl of the supernatants were spiked with 100 nmol sodium pyruvate-^13^C_3_ and 250 nmol norvaline as internal standards, afterwards the samples were dried under a gentle stream of nitrogen. For derivatization 100 µl of a methoxyamine hydrochloride solution (10 mg/1 ml pyridine) were added and the sample was shaken at 40 °C for 90 min. Afterwards 100 µl of MTBSTFA (N-(tert-butyldimethyl-silyl)-N-methyl-trifluoroacetamide containing 1% tert-butyl-dimethyl-silylchlorid) was added and the sample was heated at 70 °C for 45 min. GC-MS-analysis was performed with a QP2010 Plus or Ultra gas chromatograph/mass spectrometer (Shimadzu) equipped with a fused silica capillary column (Equity TM-5; 30 m × 0.25 mm, 0.25 μm film thickness; SUPELCO) and a quadrupole detector working with electron impact ionization at 70 eV. An aliquot of the derivatized samples was injected in 1:5 split mode at an interface temperature of 260 °C and a helium inlet pressure of 70 kPa. After sample injection, the column was first kept at 60 °C for 3 min and then developed with a temperature gradient of 10 °C min^–1^ to a final temperature of 300 °C. This temperature was held for further 3 min.

Pyruvate results were calculated relative to the pyruvate-^13^C_3_ standard (R_t_ 12.2 min), whereas all other metabolites were calculated relative to norvaline (R_t_ 17.7 min).

For qualitative sugar analysis, 50 µl of the medium were dried under a gentle stream of nitrogen. For derivatization 100 µl of a methoxyamine hydrochloride solution (10 mg/1 ml pyridine) were added and the sample was shaken at 40 °C for 90 min. Afterwards 100 µl of MSTFA (N-methyl-N (trimethylsilyl)trifluoroacetamide containing 1% trimethylchlorosilane) was added and the sample was heated at 50 °C for 45 min. GC-MS-analysis was performed as described above. Glucose, fructose, galactose, mannose and trehalose were confirmed with standard solutions.

### Spot assays

Bacterial cultures and subcultures were grown for 24 h each in 10 ml AF medium at 37 °C under anaerobic conditions without shaking. Monocultures were diluted to OD_600nm_ 0.1 in a fresh AF medium. To generate a dense bacterial lawn, monoculture inocula were diluted in LB soft agar to OD_600nm_ 0.01 and poured on a AF medium agar plate. After drying all respective other bacteria were spotted onto the bacterial lawn in duplicates in a volume of 5 μl with OD_600nm_ 0.1. Plates were incubated at 37 °C for 24 h under anaerobic conditions.

### Co-culture experiments

Monoculture inocula were prepared from a previous culture and subculture and were diluted to OD_600nm_ 0.1 in a fresh AF medium. Following, pairwise co-cultures were generated by pooling diluted inocula in a 1:1 ratio. From each co-culture 150 μl were set aside for pH measurements and determination of initial relative abundances (timepoint 0 h). The remaining co-cultures were diluted 1:10 to OD_600nm_ 0.01 and pipetted into a round-bottom 96-well plate (Nunc). Growth measurements were performed as described above for 72 h. Samples for qPCR analysis and pH measurements were taken every 24 h and the co-cultures were serially diluted 1:100 into 150 μl fresh AF medium in a new 96-well round-bottom plate to allow communities to approach a steady-state composition over ~25 bacterial generations.

### Animal experiments

C57Bl/6 mice stably associated with the OMM^12^ bacterial community were housed under germ-free conditions in flexible film isolators (North Kent Plastic Cages). Mice were supplied with autoclaved ddH_2_O and Mouse-Breeding complete feed for mice (Ssniff) ad libitum. For the experiment shown in Fig. 5C, 12 weeks old male mice were used and sacrificed by cervical dislocation at ZG 10. Intestinal content was harvested, weighed, and frozen at –20 °C before DNA extraction. For the experiment shown in Fig. S20, 7 day old male and female mice were used and sacrificed by decapitation at ZG 4. Intestinal sections were frozen at –20 °C before DNA extraction.

### DNA extraction for co-culture samples

DNA extraction was performed in the 96-well format using the PureLink^TM^ Pro 96 genomic DNA Kit (Invitrogen) following the corresponding lysis protocol for Gram-positive bacterial cells using lysozyme and proteinase K.

### DNA extraction from intestinal contents and in vitro community samples

gDNA extraction using a phenol-chloroform based protocol was performed as described previously [25]. Fecal pellet or cecal content was resuspended in 500 µl extraction buffer (200 mM Tris-HCl, 200 mM NaCl, 20 mM EDTA in ddH_2_O, pH 8, autoclaved), 210 µl 20 % SDS and 500 µl phenol:chloroform:isoamylalcohol (25:24:1, pH 7.9). Furthermore, 0.1 mm-diameter zirconia/silica beads (Roth) were added. Bacteria were lysed with a bead beater (TissueLyser LT, Qiagen) for 4 min, 50 Hz. After centrifugation (14,000 x g, 5 min, RT), the aqueous phase was transferred into a new tube, 500 µl phenol:chloroform:isoamylalcohol (25:24:1, pH 7.9) were added and again spun down. The resulting aqueous phase was gently mixed with 1 ml 96 % ethanol and 50 µl of 3 M sodium acetate by inverting. After centrifugation (30 min, 14,000 x g, 4 °C), the supernatant was discarded and the gDNA pellet was washed with 500 µl ice-cold 70 % ethanol and again centrifuged (14,000 x g, 4 °C; 15 min). The resulting gDNA pellet was resuspended in 100 µl Tris-HCL pH 8. Subsequently, gDNA was purified using the the NucleoSpin gDNA clean-up kit (Macherey–Nagel) and stored at –20 °C.

### Quantitative PCR of bacterial 16 S rRNA genes

Quantitative PCR was performed as described previously [23]. 5 ng gDNA was used as a template for qPCR. Only for samples from infant mice (Fig. S20) 50 ng were used. Strain-specific 16S rRNA primers and hydrolysis probes were used for amplification. Standard curves were determined using linearized plasmids containing the 16S rRNA gene sequence of the individual strains. The standard specific efficiency was then used for absolute quantification of 16S rRNA gene copy numbers of individual strains.

### Determination of co-culture outcomes

Quantitative 16S rRNA copy numbers from the measurement endpoint of three independent co-culture experiments were determined by qPCR. Co-culture outcomes (positive, neutral, or negative) were determined by calculating the individual abundance ratio for each strain in co-culture relative to monoculture. Therefore, the strain-specific absolute abundance at 72 h in all pairwise co-cultures was divided by the strain-specific absolute abundance at 72 h in monoculture $( {{{{{{{{\mathrm{r}}}}}}}}_{{{{{{{{\mathrm{i,bm}}}}}}}}}\frac{{m_{i,co}(t72h)}}{{m_{i,mono}(t72h)}}})$ for every individual experiment. Following, the mean abundance ratio from all individual experiments (*n* = 3 per strain combination) was calculated. Significance was determined using a two-sided *t*-test.

### Community experiments

Monoculture inocula were prepared from a previous culture and subculture and were diluted to OD_600nm_ 0.1 in fresh AF medium. Following, the community inoculum with equivalent ratios of all 12 strains was generated from this dilution. The inoculum was distributed to 24-well plates, thereby diluting the inoculum 1:10 to 1 ml fresh AF medium, resulting in a starting OD_600nm_ of 0.01. 24-well plates were incubated at 37 °C without shaking under anaerobic conditions. Every 24 h for ten days samples were taken for qPCR analysis, OD measurement and pH measurement, and cultures were diluted 1:100 in 1 ml fresh-AF medium.

### Malate and L-Methionine uptake measurements

Uptake of ^14^C-malate and ^3^H-L-methionine by *E. faecalis* KB1 was determined in principle as previously described [67]. Briefly, *E. faecalis* was grown anaerobically in LB medium with 40 mM malate or L-methionine respectively and harvested in mid-log phase. Cells were centrifuged, washed twice with 50 mM Tris-HCl buffer (pH 7.4) containing 10 mM MgCl_2_ and resuspended in 50 mM Tris-maleate buffer (pH 7.2) containing 5 mM MgCl_2_, thereby adjusting the cell suspension to an OD_600_ of 10. For malate transport assays, this cell suspension was diluted 1:10 with 50 mM Tris-maleate buffer (pH 7.2) containing 5 mM MgCl_2_ and 1% (w/v) peptone. For L-methionine transport assays, this cell suspension was diluted 1:10 with 50 mM Tris-maleate buffer (pH 7.2) containing 5 mM MgCl_2_. Uptake of ^14^C-malate (55 mCi mmol^–1^ [Biotrend]) and ^3^H-L-methionine (55 Ci mmol^–1^ [Biotrend]) was measured at a total substrate concentration of 10 µM at 18 °C. At various time intervals, the transport was terminated by the addition of stop buffer (100 mM potassium phosphate buffer, pH 6.0, 100 mM LiCl), followed by rapid filtration through membrane filters (MN gf-5 0.4 µm; Macherey–Nagel). The filters were dissolved in 5 ml of scintillation fluid (MP Biomedicals), and radioactivity was determined in a liquid scintillation analyzer (PerkinElmer). Total protein content of *E. faecalis* cells in relation to OD_600_ was determined with a suspension of lysed cells as described before [68]. The effects of protonophores and ionophores were tested after preincubation of cells in 50 mM potassium phosphate buffer (pH 7.2) containing 5 mM MgCl_2_ and 1% peptone, supplemented with 20 µM carbonyl cyanide m-chlorophenylhydrazone (CCCP), 2 mM 2,4-dinitrophenol (DNP), 10 µM nonactin, 6 µM nigericin, 2 µM valinomycin or dimethyl sulfoxide (DMSO, as a control) at room temperature for 10 min.

### Data analysis and figures

Data was analyzed using R Studio (Version 1.4.1103). Heatmaps were generated using the R *pheatmap* package (https://github.com/raivokolde/pheatmap). Plots were generated using the R *ggplot2* package [69] and *ggpubR* package (https://github.com/kassambara/ggpubr). Figures were partly generated using BioRender (https://biorender.com) and Adobe Illustrator CC (Adobe Inc.).

## Supplementary information

Supplemental material

SI data table

SI data metabolic models

SI data file

## References

[1] Gilbert JA, Blaser MJ, Caporaso JG, Jansson JK, Lynch SV, Knight R (2018). Current understanding of the human microbiome. Nat Med.

[2] Forster SC, Kumar N, Anonye BO, Almeida A, Viciani E, Stares MD (2019). A human gut bacterial genome and culture collection for improved metagenomic analyses. Nat Biotechnol.

[3] Amor D, Ratzke C, Gore J Transient invaders can induce shifts between alternative stable states of microbial communities. Sci Adv. 2020 10.1126/sciadv.aay8676. 10.1126/sciadv.aay8676 PMC7030923 32128414

[4] Blasche S, Kim Y, Mars RAT, Machado D, Maansson M, Kafkia E (2021). Metabolic cooperation and spatiotemporal niche partitioning in a kefir microbial community. Nat Microbiol.

[5] Faith JJ, Guruge JL, Charbonneau M, Subramanian S, Seedorf H, Goodman AL (2013). The long-term stability of the human gut microbiota. Science.

[6] Coyte KZ, Schluter J, Foster KR (2015). The ecology of the microbiome: networks, competition, and stability. Science.

[7] Gralka M, Szabo R, Stocker R, Cordero OX (2020). Trophic interactions and the drivers of microbial community assembly. Curr Biol.

[8] Granato ET, Meiller-Legrand TA, Foster KR (2019). The evolution and ecology of bacterial warfare. Curr Biol.

[9] Caballero S, Kim S, Carter RA, Leiner IM, Susac B, Miller L (2017). Cooperating commensals restore colonization resistance to vancomycin-resistant Enterococcus faecium. Cell Host Microbe.

[10] Gutiérrez N, Garrido D Species deletions from microbiome consortia reveal key metabolic interactions between gut microbes. mSystems. 2019 10.1128/mSystems.00185-19. 10.1128/mSystems.00185-19 PMC6635622 31311843

[11] Ratzke C, Barrere J, Gore J (2020). Strength of species interactions determines biodiversity and stability in microbial communities. Nat Ecol Evol.

[12] Kim S, Covington A, Pamer EG (2017). The intestinal microbiota: antibiotics, colonization resistance, and enteric pathogens. Immunol Rev.

[13] Kreuzer M, Hardt WD (2020). How food affects colonization resistance against enteropathogenic bacteria. Annu Rev Microbiol.

[14] Lloyd-Price J, Arze C, Ananthakrishnan AN, Schirmer M, Avila-Pacheco J, Poon TW (2019). Multi-omics of the gut microbial ecosystem in inflammatory bowel diseases. Nature.

[15] Pereira FC, Wasmund K, Cobankovic I, Jehmlich N, Herbold CW, Lee KS (2020). Rational design of a microbial consortium of mucosal sugar utilizers reduces Clostridiodes difficile colonization. Nat Commun.

[16] Freilich S, Kreimer A, Meilijson I, Gophna U, Sharan R, Ruppin E (2010). The large-scale organization of the bacterial network of ecological co-occurrence interactions. Nucleic Acids Res.

[17] Shoaie S, Karlsson F, Mardinoglu A, Nookaew I, Bordel S, Nielsen J Understanding the interactions between bacteria in the human gut through metabolic modeling. Sci Rep. 2013 10.1038/srep02532. 10.1038/srep02532 PMC3755282 23982459

[18] Shoaie S, Ghaffari P, Kovatcheva-Datchary P, Mardinoglu A, Sen P, Pujos-Guillot E (2015). Quantifying diet-induced metabolic changes of the human gut microbiome. Cell Metab.

[19] Biggs MB, Medlock GL, Moutinho TJ, Lees HJ, Swann JR, Kolling GL (2017). Systems-level metabolism of the altered Schaedler flora, a complete gut microbiota. ISME J.

[20] Medlock G, Carey M, McDuffie D, Mundy M, Giallourou N, Swann J, et al. Inferring metabolic mechanisms of interaction within a defined gut microbiota. Cell Syst. 2018 10.1016/j.cels.2018.08.003. 10.1016/j.cels.2018.08.003 PMC6166237 30195437

[21] Venturelli OS, Carr AC, Fisher G, Hsu RH, Lau R, Bowen BP (2018). Deciphering microbial interactions in synthetic human gut microbiome communities. Mol Syst Biol.

[22] Clark R, Connors B, Stevenson D, Hromada S, Hamilton J, Amador-Noguez D, et al. Design of synthetic human gut microbiome assembly and function. bioRxiv. 2020 10.1101/2020.08.19.241315. 10.1038/s41467-021-22938-y PMC8166853 34059668

[23] Brugiroux S, Beutler M, Pfann C, Garzetti D, Ruscheweyh H, Ring D, et al. Genome-guided design of a defined mouse microbiota that confers colonization resistance against Salmonella enterica serovar Typhimurium. Nat Microbiol. 2016:10.1038/nmicrobiol.2016.215. 10.1038/nmicrobiol.2016.215 27869789

[24] Studer N, Desharnais L, Beutler M, Brugiroux S, Terrazos MA, Menin L (2016). Functional intestinal bile acid 7alpha-dehydroxylation by clostridium scindens associated with protection from clostridium difficile infection in a gnotobiotic mouse model. Front Cell Infect Microbiol.

[25] Herp S, Brugiroux S, Garzetti D, Ring D, Jochum LM, Beutler M (2019). Mucispirillum schaedleri antagonizes Salmonella virulence to protect mice against colitis. Cell Host Microbe.

[26] Eberl C, Ring D, Munch PC, Beutler M, Basic M, Slack EC (2019). Reproducible colonization of germ-free mice with the oligo-mouse-microbiota in different animal facilities. Front Microbiol.

[27] Garzetti D, Brugiroux S, Bunk B, Pukall R, McCoy KD, Macpherson AJ, et al. High-quality whole-genome sequences of the oligo-mouse-microbiota bacterial community. Genome Announc. 2017;5 10.1128/genomeA.00758-17. 10.1128/genomeA.00758-17 PMC5646386 29051233

[28] Lagkouvardos I, Pukall R, Abt B, Foesel B, Stecher B, Clavel T The Mouse Intestinal Bacterial Collection (miBC) provides host-specific insight into cultured diversity and functional potential of the gut microbiota. Nat Microbiol. 2016:10.1038/nmicrobiol.2016.131. 10.1038/nmicrobiol.2016.131 27670113

[29] Bolsega S, Basic M, Smoczek A, Buettner M, Eberl C, Ahrens D (2019). Composition of the intestinal microbiota determines the outcome of virus-triggered colitis in mice. Front Immunol.

[30] Kuczma MP, Szurek EA, Cebula A, Chassaing B, Jung YJ, Kang SM (2020). Commensal epitopes drive differentiation of colonic Tregs. Sci Adv.

[31] Nowosad CR, Mesin L, Castro TBR, Wichmann C, Donaldson GP, Araki T (2020). Tunable dynamics of B cell selection in gut germinal centres. Nature.

[32] Marion S, Desharnais L, Studer N, Dong Y, Notter MD, Poudel S (2020). Biogeography of microbial bile acid transformations along the murine gut. J Lipid Res.

[33] Cintas LM, Casaus P, Holo H, Hernandez PE, Nes IF, Havarstein LS (1998). Enterocins L50A and L50B, two novel bacteriocins from Enterococcus faecium L50, are related to staphylococcal hemolysins. J Bacteriol.

[34] Blin K, Shaw S, Steinke K, Villebro R, Ziemert N, Lee SY (2019). antiSMASH 5.0: updates to the secondary metabolite genome mining pipeline. Nucleic Acids Res.

[35] Zimmermann J, Kaleta C, Waschina S (2021). gapseq: informed prediction of bacterial metabolic pathways and reconstruction of accurate metabolic models. Genome Biol.

[36] Caspi R, Billington R, Keseler IM, Kothari A, Krummenacker M, Midford PE (2020). The MetaCyc database of metabolic pathways and enzymes - a 2019 update. Nucleic Acids Res.

[37] Berry D, Widder S (2014). Deciphering microbial interactions and detecting keystone species with co-occurrence networks. Front Microbiol.

[38] Goberna M, Verdu M (2016). Predicting microbial traits with phylogenies. ISME J.

[39] Langille M, Zaneveld J, Caporaso J, McDonald D, Knights D, Reyes J, et al. Predictive functional profiling of microbial communities using 16S rRNA marker gene sequences. Nat Biotechnol. 2013 10.1038/nbt.2676. 10.1038/nbt.2676 PMC3819121 23975157

[40] Walker AW, Duncan SH, McWilliam Leitch EC, Child MW, Flint HJ (2005). pH and peptide supply can radically alter bacterial populations and short-chain fatty acid ratios within microbial communities from the human colon. Appl Environ Microbiol.

[41] Cotter PD, Ross RP, Hill C (2013). Bacteriocins - a viable alternative to antibiotics?. Nat Rev Microbiol.

[42] Ubeda C, Taur Y, Jenq R, Equinda M, Son T, Samstein M, et al. Vancomycin-resistant Enterococcus domination of intestinal microbiota is enabled by antibiotic treatment in mice and precedes bloodstream invasion in humans. J Clin Investig. 2020 10.1172/JCI43918. 10.1172/JCI43918 PMC2993598 21099116

[43] Mortera P, Espariz M, Suarez C, Repizo G, Deutscher J, Alarcon S (2012). Fine-tuned transcriptional regulation of malate operons in Enterococcus faecalis. Appl Environ Microbiol.

[44] Hughes KR, Schofield Z, Dalby MJ, Caim S, Chalklen L, Bernuzzi F (2020). The early life microbiota protects neonatal mice from pathological small intestinal epithelial cell shedding. FASEB J.

[45] Estrela S, Sanchez-Gorostiaga A, Vila J, Sanchez A Nutrient dominance governs the assembly of microbial communities in mixed nutrient environments. preprint. 2020 10.1101/2020.08.06.239897. 10.7554/eLife.65948 PMC8057819 33877964

[46] Rettedal EA, Gumpert H, Sommer MO (2014). Cultivation-based multiplex phenotyping of human gut microbiota allows targeted recovery of previously uncultured bacteria. Nat Commun.

[47] Tramontano M, Andrejev S, Pruteanu M, Klunemann M, Kuhn M, Galardini M (2018). Nutritional preferences of human gut bacteria reveal their metabolic idiosyncrasies. Nat Microbiol.

[48] Berry D, Stecher B, Schintlmeister A, Reichert J, Brugiroux S, Wild B (2013). Host-compound foraging by intestinal microbiota revealed by single-cell stable isotope probing. Proc Natl Acad Sci USA.

[49] Ottman N, Davids M, Suarez-Diez M, Boeren S, Schaap PJ, Martins Dos Santos VAP, et al. Genome-scale model and omics analysis of metabolic capacities of Akkermansia muciniphila reveal a preferential mucin-degrading lifestyle. Appl Environ Microbiol. 2017;83 10.1128/AEM.01014-17. 10.1128/AEM.01014-17 PMC5583483 28687644

[50] Seeman T Prokka: rapid prokaryotic genome annotation. Bioinformatics. 2014:10.1093/bioinformatics/btu153. 10.1093/bioinformatics/btu153 24642063

[51] Pruesse E, Peplies J, Glockner FO (2012). SINA: accurate high-throughput multiple sequence alignment of ribosomal RNA genes. Bioinformatics.

[52] Stamatakis A (2014). RAxML version 8: a tool for phylogenetic analysis and post-analysis of large phylogenies. Bioinformatics.

[53] Revell L phytools: an R package for phylogenetic comparative biology (and other things). Methods Ecol Evol. 2012 10.1111/j.2041-210X.11.00169.x.

[54] Letunic I, Bork P (2007). Interactive Tree Of Life (iTOL): an online tool for phylogenetic tree display and annotation. Bioinformatics.

[55] Saier MH, Reddy VS, Tamang DG, Vastermark A (2014). The transporter classification database. Nucleic Acids Res.

[56] Marinos G, Kaleta C, Waschina S (2020). Defining the nutritional input for genome-scale metabolic models: a roadmap. PLoS One.

[57] Wood T Some applications of paper chromatography to the examination of meat extract. Sci Food Agric. 1956;7.

[58] Biosciences BBD, Biosciences BBD Bacto TM Beef Extract, Desiccated. Technical Manual BD Biosciences – Advanced Bioprocessing. 4: BD Biosciences – Advanced Bioprocessing; 2015. p. 30.

[59] Freshney RI culture of animal cells: a manual of basic technique and specialized applications: Hoboken: John Wiley & Sons; 2016.

[60] Petruschke H, Schori C, Canzler S, Riesbeck S, Poehlein A, Daniel R (2021). Discovery of novel community-relevant small proteins in a simplified human intestinal microbiome. Microbiome.

[61] Kessner D, Chambers M, Burke R, Agus D, Mallick P (2008). ProteoWizard: open source software for rapid proteomics tools development. Bioinformatics.

[62] Smith C, Want E, O’Maille G, Abagyan R, Siuzdak G XCMS: processing mass spectrometry data for metabolite profiling using nonlinear peak alignment, matching, and identification. Anal Chem. 2006 10.1021/ac051437y. 10.1021/ac051437y 16448051

[63] Wishart DS, Feunang YD, Marcu A, Guo AC, Liang K, Vazquez-Fresno R (2018). HMDB 4.0: the human metabolome database for 2018. Nucleic Acids Res.

[64] Tsugawa H, Cajka T, Kind T, Ma Y, Higgins B, Ikeda K (2015). MS-DIAL: data-independent MS/MS deconvolution for comprehensive metabolome analysis. Nat Methods.

[65] Haug K, Cochrane K, Nainala VC, Williams M, Chang J, Jayaseelan KV (2020). MetaboLights: a resource evolving in response to the needs of its scientific community. Nucleic Acids Res.

[66] Han J, Lin K, Sequeira C, Borchers CH (2015). An isotope-labeled chemical derivatization method for the quantitation of short-chain fatty acids in human feces by liquid chromatography-tandem mass spectrometry. Anal Chim Acta.

[67] Mokhtari A, Blancato VS, Repizo GD, Henry C, Pikis A, Bourand A (2013). Enterococcus faecalis utilizes maltose by connecting two incompatible metabolic routes via a novel maltose 6'-phosphate phosphatase (MapP). Mol Microbiol.

[68] Bradford MM (1976). A rapid and sensitive method for the quantitation of microgram quantities of protein utilizing the principle of protein-dye binding. Anal Biochem.

[69] Wickham H (2016). ggplot2: elegant graphics for data analysis.

