# Supplementary material for: In vitro interaction network of a synthetic gut bacterial community
Source: ISME J. 2021 Dec 2;16(4):1095–109. doi: 10.1038/s41396-021-01153-z (PMC8941000; doi:10.1038/s41396-021-01153-z)
Supplement: Supplementary file 1 — Supplemental material [file 41396_2021_1153_MOESM1_ESM.pdf]

Supplemental Information

Supplemental Figures

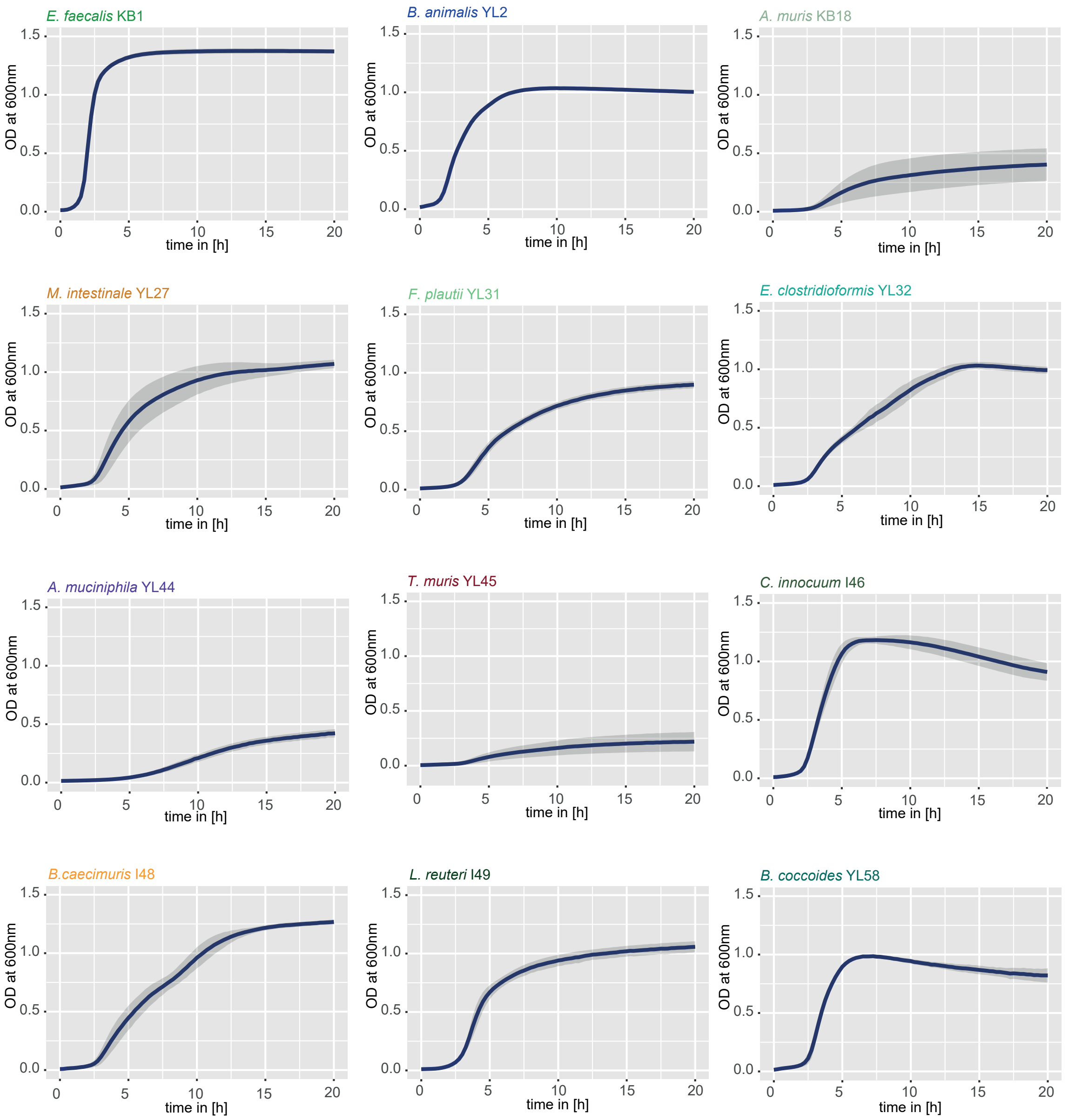

Fig. S1. Growth characteristics of individual strains in monoculture. Growth in AF medium was monitored at OD 600nm, mean (blue line) and SD (grey) of three independent experiments is shown. Growth rates were extrapolated from growth in the exponential phase (Tab. S2). All strains grew to stationary phase within 20 hours and strain specific behavior in AF medium can be observed. Interestingly, strains *E. clostridioformis* YL32 and *B. caecimuris* I48 show indications of a diauxic growth behavior. Further, strains *C. innocuum* I46 and *B. coccoides* YL58 show a decrease in OD 600nm after stationary phase is reached.

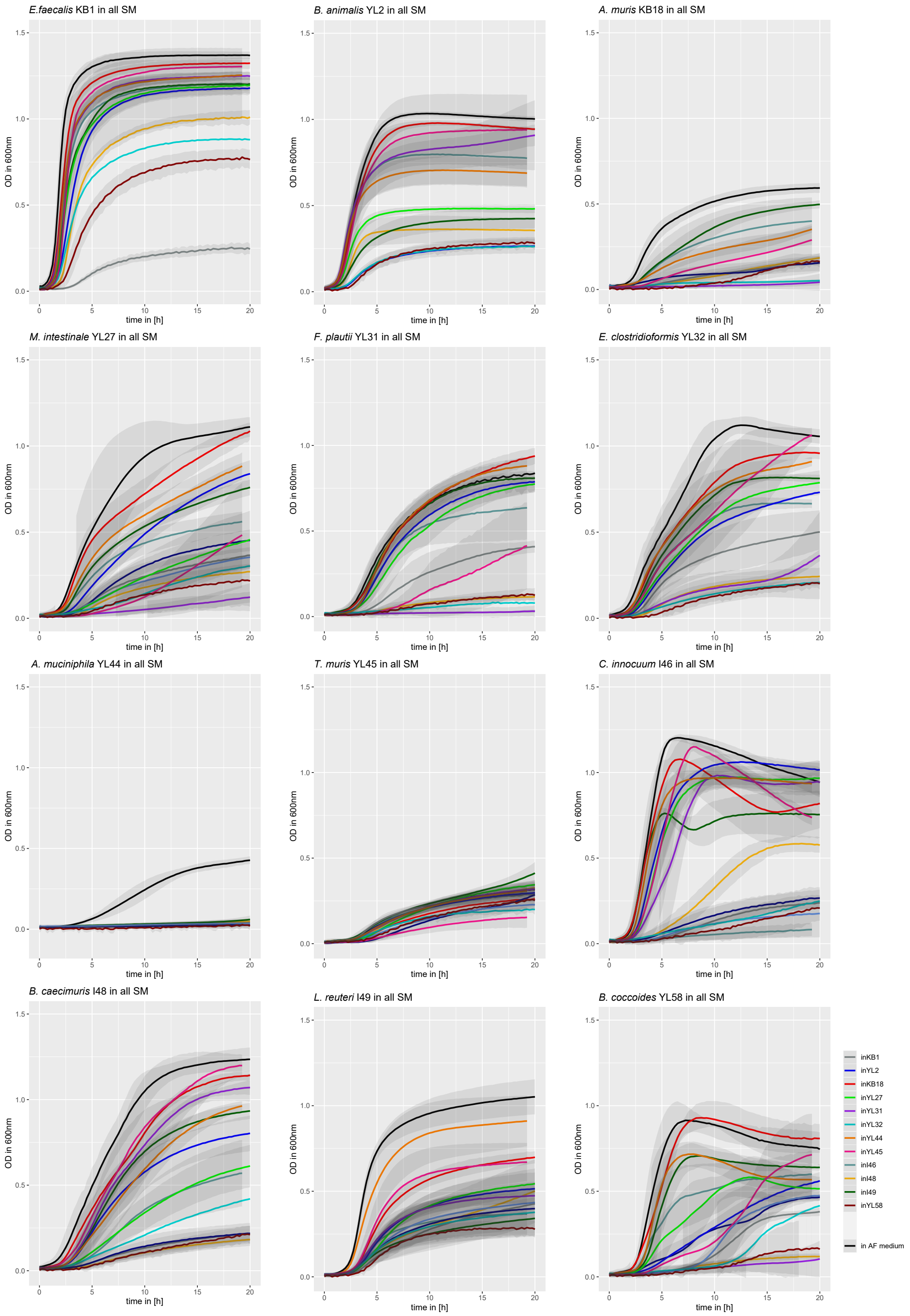

Fig. S2. Growth curves of individual strains in SM of all OMM^{12} bacteria. Growth of all individual monocultures was monitored in fresh AF medium (black) and in SM of individual OMM^{12} strains (colored lines) over 20 hours at OD 600nm. The mean of three indepedent experiments is shown with the corresponding SD (grey).

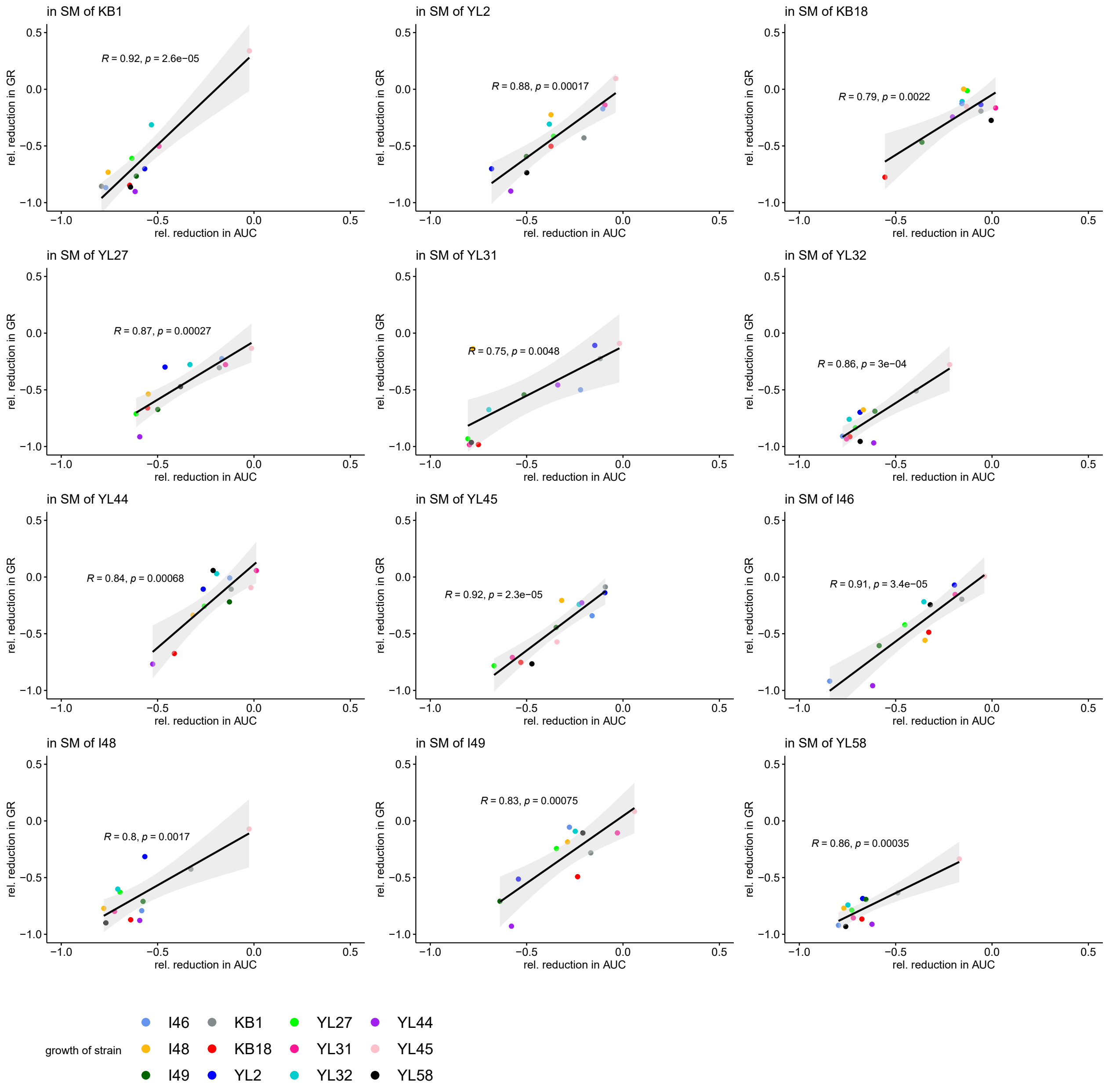

Fig. S 3. Correlation of reduction in growth rate and reduction in AUC. The reduction of growth rate and AUC in SM relative to the corresponding fresh AF medium control was calculated for all strains of the OMM^{12} consortium from data shown in **Fig S2**. Relative coefficients were then correlated and show a negative linear relation for all individual strains, indicating that both growth rate and AUC in a specific SM are decreased to the same extent. The corresponding values for the individual strains are shown in colors, the linear fit (black) with the standard error (grey).

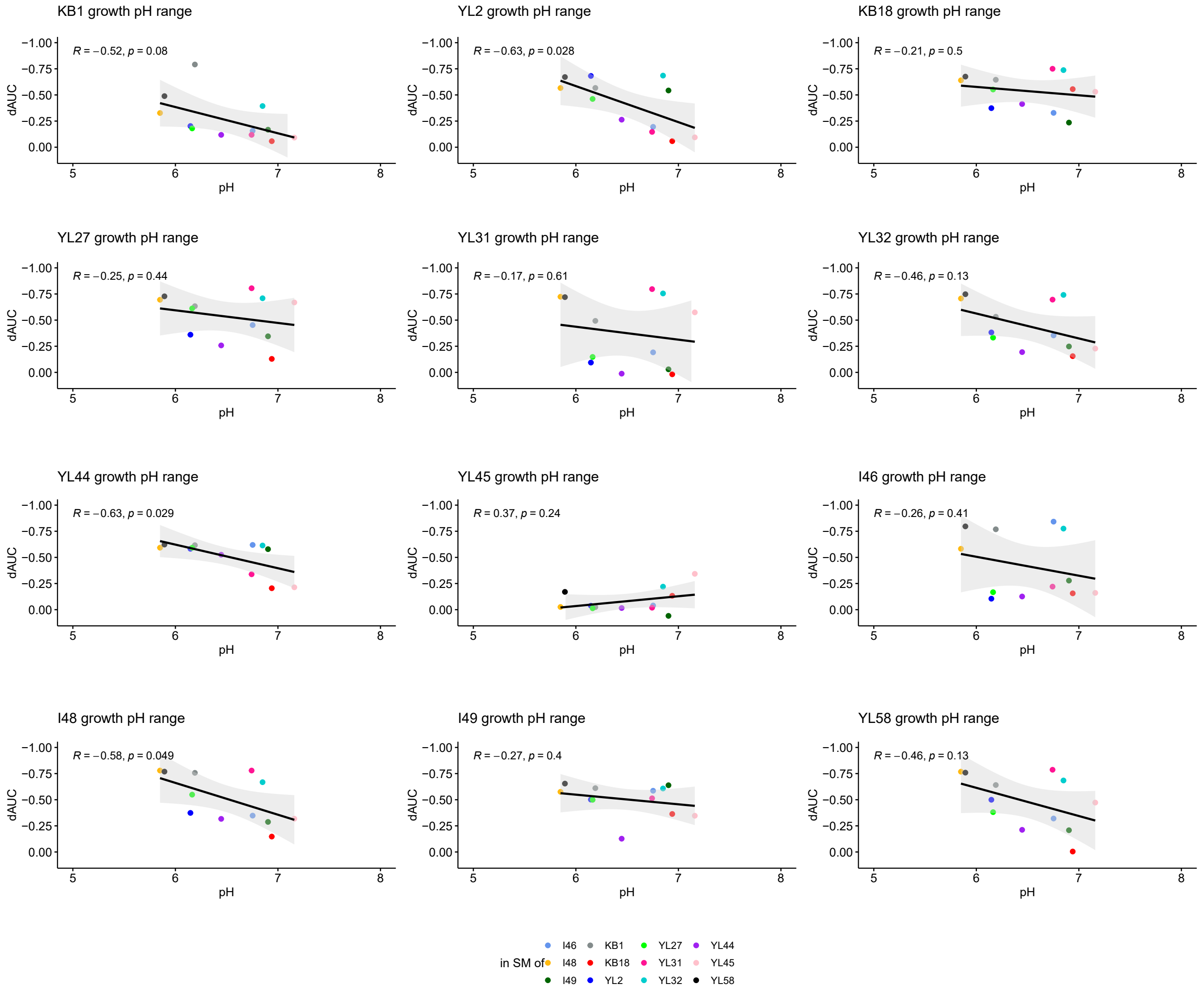

Fig. S4. Correlation of reduction in AUC with the pH of the SM of a specific strain. Correlation of the inhibition of growth in a SM (d_{AUC}) with the mean pH of the individual SM for each strain revealed that growth inhibition does not directly correlate with the pH for all strains. Only *B. animalis* YL2, *A. muciniphila* YL44 and *B. caecimuris* I48 showed a significant negative correlation (R < - 0.5, p < 0.05) between growth inhibition and pH with stronger inhibition at more acidic pH ranges. The corresponding values for the individual strains are shown in colors, the linear fit (black) is depicted with the SD (grey).

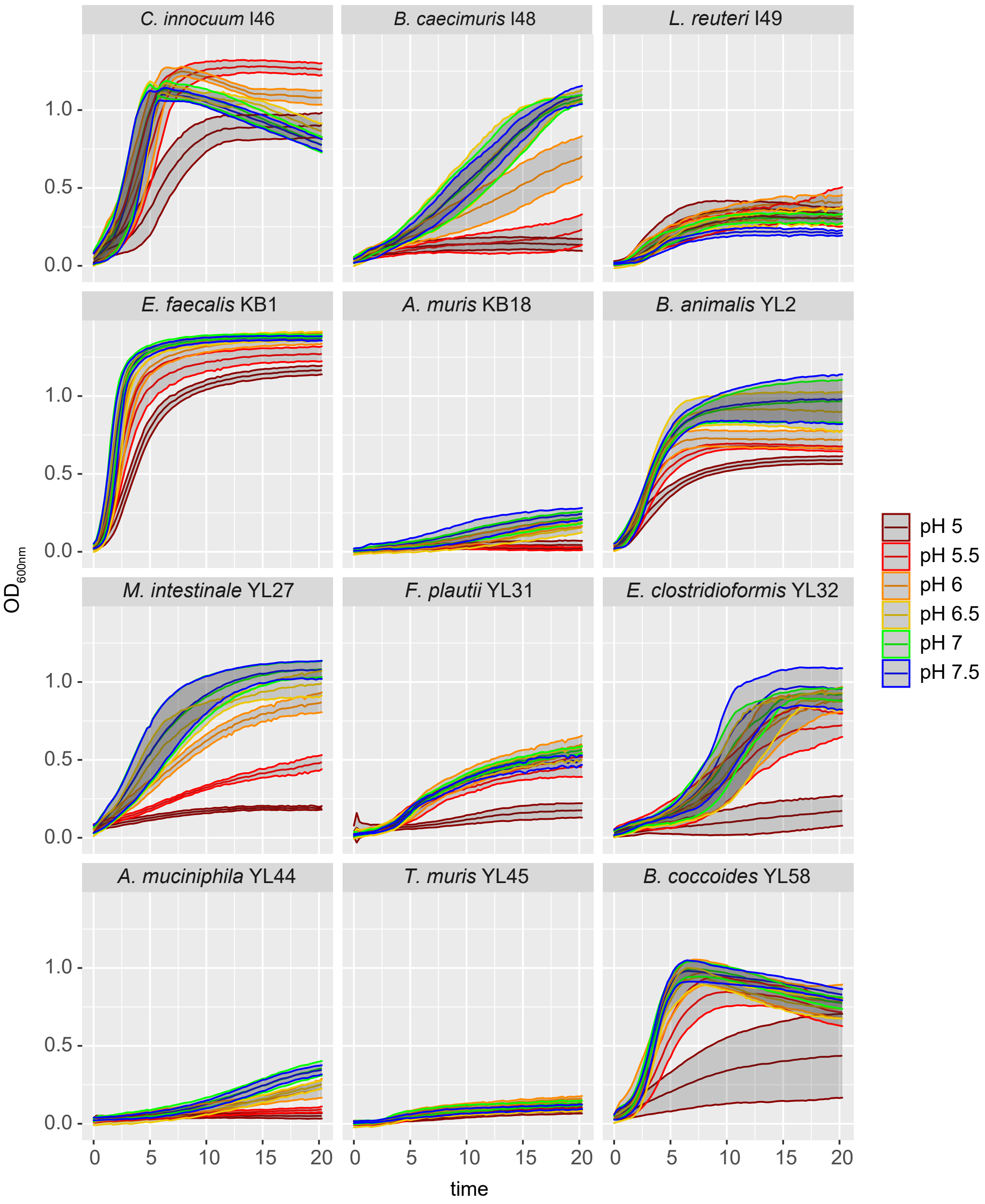

Fig. S5. Growth curves of individual OMM^{12} strains in AF media at different pH. Growth of all individual monocultures was monitored in AF medium at pH 5.0 to pH 7.5 (colored lines) over 20 hours at OD 600nm. The mean of three independent experiments is shown with the corresponding SD (grey).

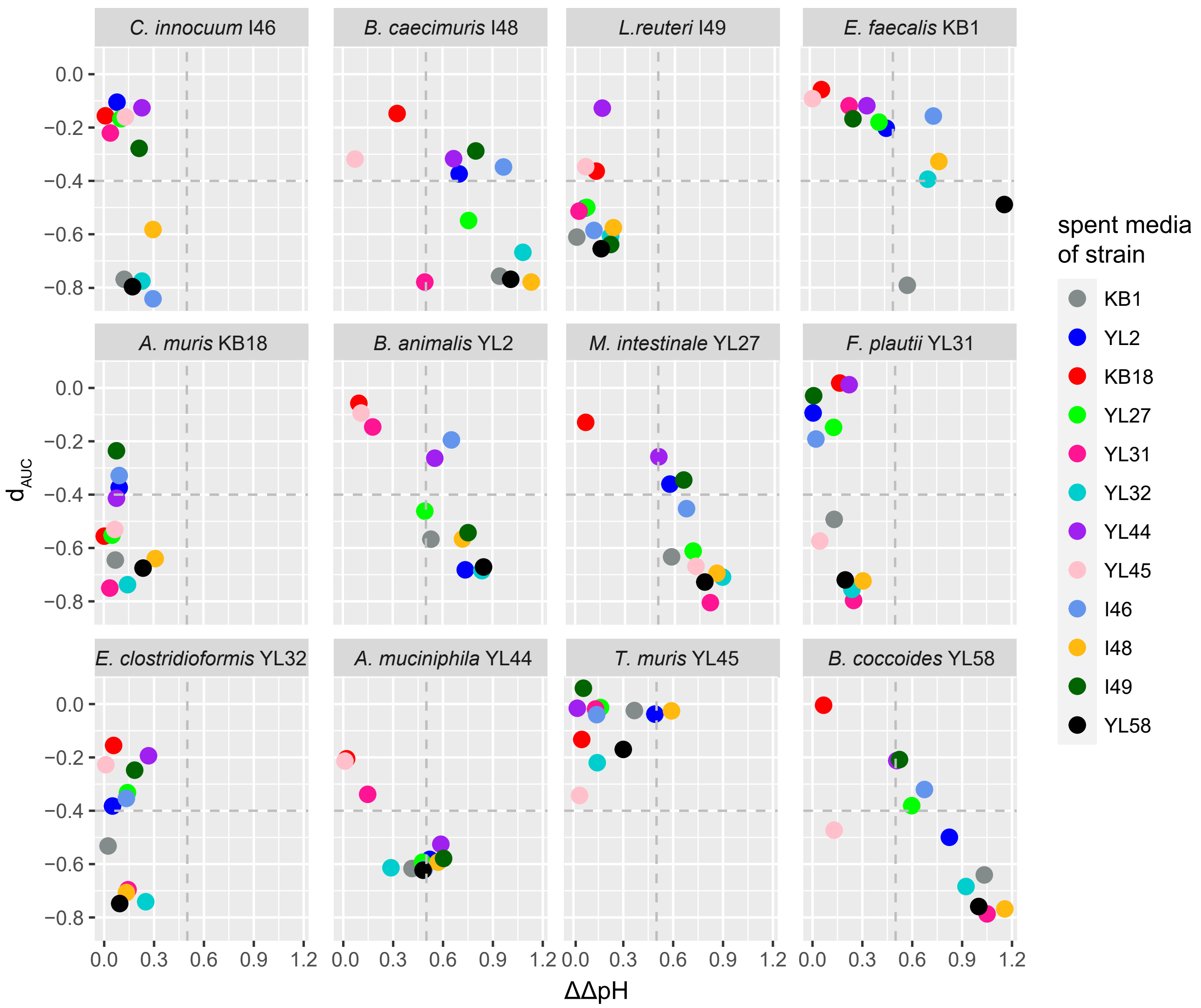

Fig. S6. Relationship between AUC decrease in SM and change in pH profiles. The inhibition factor d_{AUC} derived from spent media experiments is plotted against the corresponding $\Delta\Delta pH$ value. Each plot shows the parameters of every individual strain in the spent media (SM) of all the other strains (colored dots). The factor $\Delta\Delta pH$ depicts the Euclidean distance between the ΔpH_{SM} (after growth in fresh AF medium) and the corresponding ΔpH_{DSM} (after growth in a respective SM) and is a measure how the pH profile of a individual strain changes in the environment of a specific SM in comparison to fresh medium (see supplemental text). Cases where overall growth is only slightly influenced in SM ($d_{AUC} > -0.4$), but the corresponding ΔpH changed strongly ($\Delta\Delta pH > 0.5$, upper right quadrant) might indicate a drastic change in metabolic behavior of a strain due to the altered environmental conditions in SM. For example, the $\Delta\Delta pH$ of *E. faecalis* KB1 in the SM of *C. innocuum* I46 indicates (grey circle), that here the metabolic behavior of KB1 is altered in comparison to fresh AF medium. While KB1 strongly acidifies the neutral fresh culture medium($pH = 7.0$) to $pH_{SM,\ KB1} = 6.19$ ($\Delta pH_{SM} =$ - 0.81), the neutral SM of I46 ($pH_{SM,\ I46} = 6.75$) is not distinctly acidified after KB1 growth ($pH_{DSM} = 6.68$, corresponding to $\Delta pH_{DSM} =$ - 0.07 and therefore $\Delta\Delta pH = 0.74$).

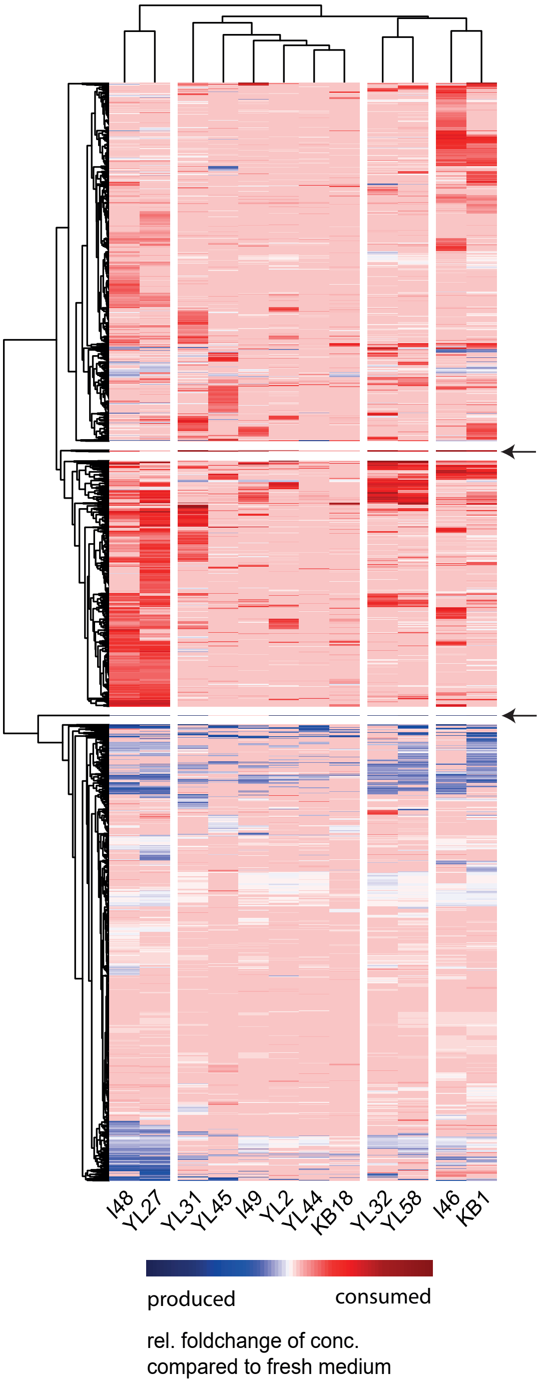

Fig. S7. Untargeted metabolomics analysis of SM. Metabolomic profiles after bacterial growth to stationary phase in AF medium were determined by untargeted MS (methods). All metabolomic features that significantly changed for at least one of the twelve strains are shown (rows). Levels decreased (red) and levels increased (blue) compared to fresh AF medium as determined by the relative foldchange. Hierarchical clustering of strain specific profiles as well as measured metabolomic features reveal profile similarities between phylogenetically related strains. Black arrows indicate two separated clusters of metabolomic features that are produced and consumed by most bacterial strains.

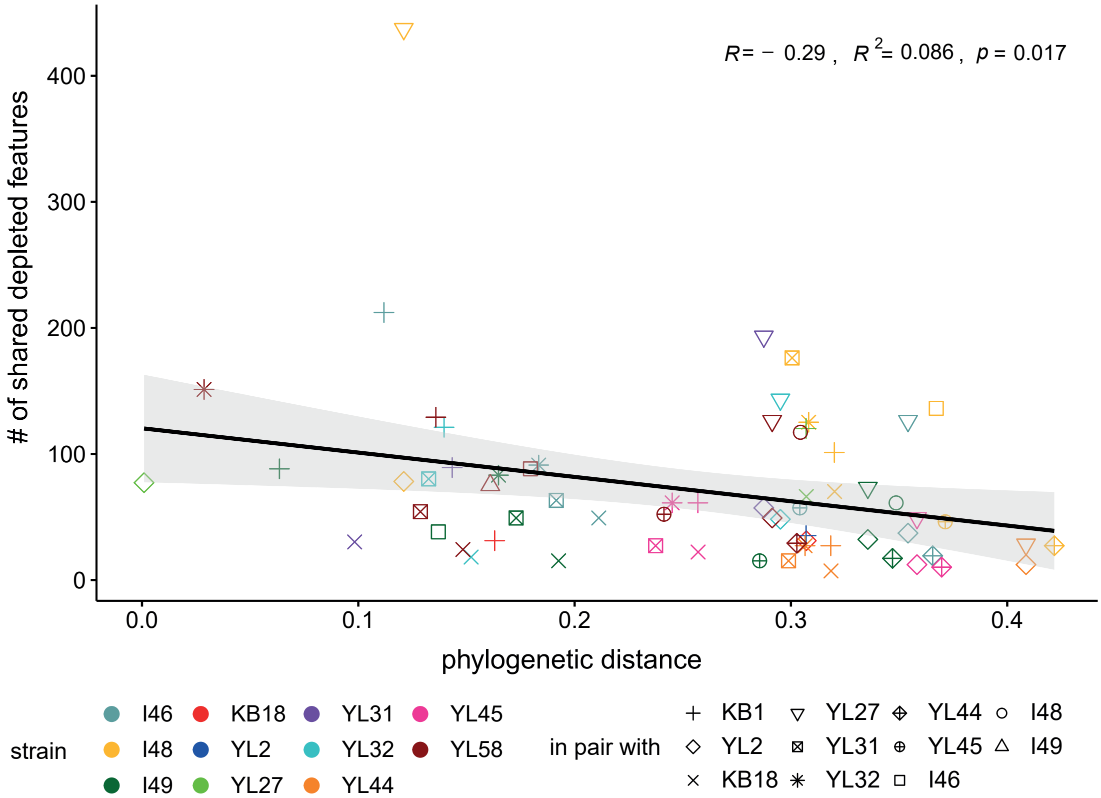

Fig. S8. Correlation of overlap in substrate depletion profiles with phylogenetic distance. Correlating the phylogenetic distance between the individual strains (based on 16S rRNA gene sequences) with the number of shared depleted metabolic features in AF medium showed that phylogenetically similar strains of the consortium have a higher substrate overlap than phylogenetically distant strains. The corresponding values for the individual strain pairs are shown in colors and shapes, the linear fit is depicted (black line) with the standard error (grey).

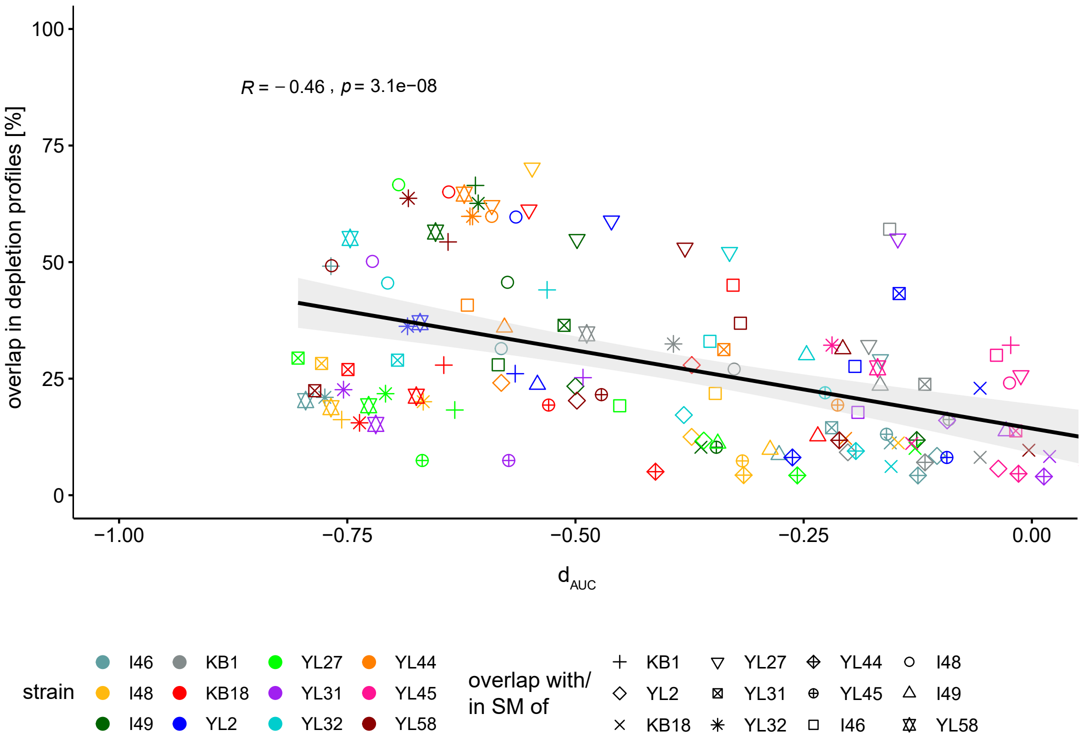

Fig. S9. Correlation of overlap in substrate depletion profiles with inhibition of growth in SM. Correlating the pairwise overlap in depleted metabolic features in AF medium with the inhibition of growth in the corresponding SM (d_{AUC}) revealed that overlap in depletion profiles is correlated with growth inhibition in the corresponding SM. The corresponding values for the individual strain pairs are shown in colors and shapes, the linear fit is depicted (black line) with the standard error (grey).

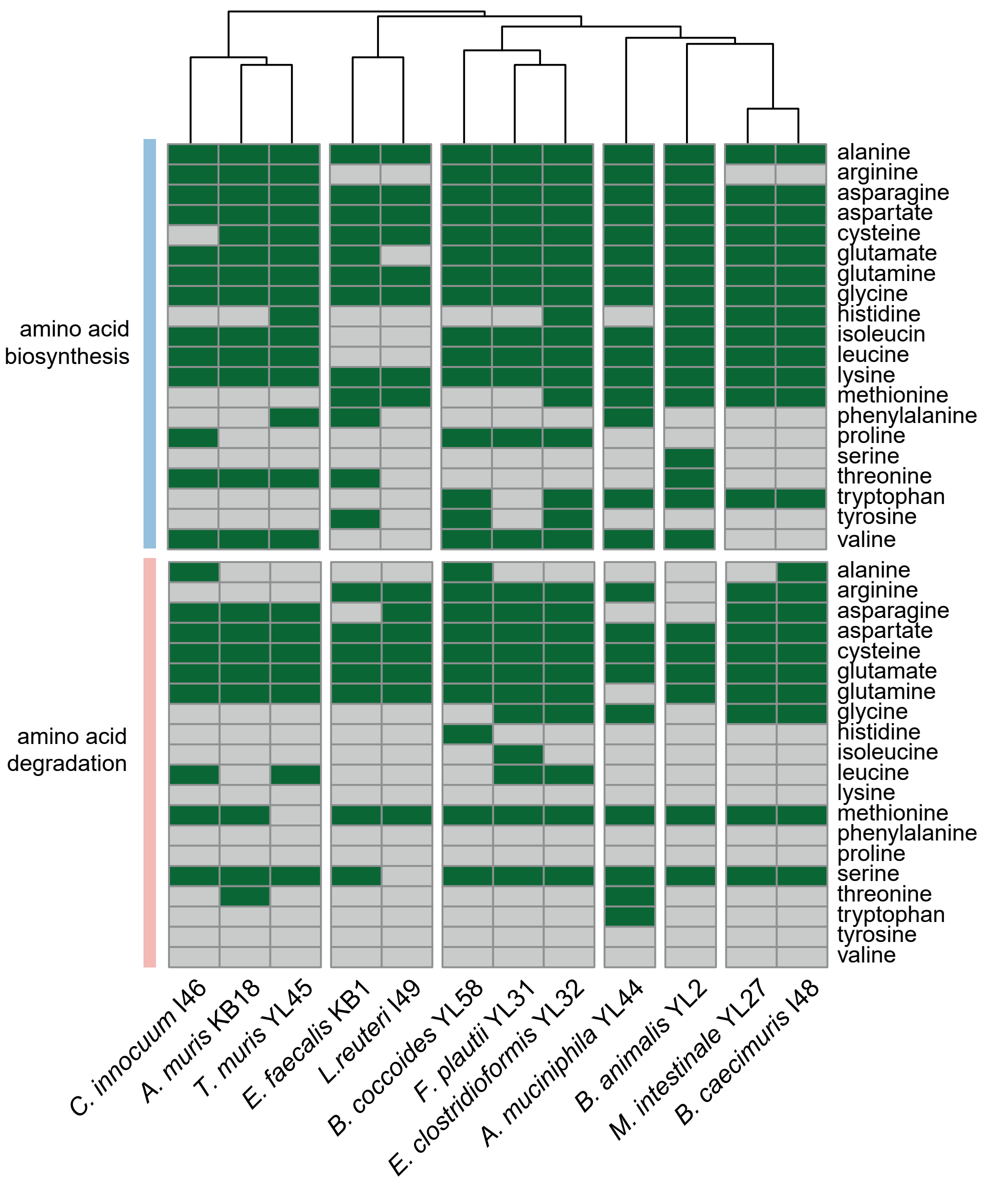

Fig. S10. Genome-informed potential of amino acid biosynthesis and degradation. OMM^{12} metabolic models were reconstructed using gapseq and gapseq output was screened for a hand-curated set of pathways to determine the strains' potential to synthesize and degrade amino acids (Methods). Multiple pathways corresponding to the same function were grouped together according to the MetaCyc pathway ontology (SI data table 2) and pathway utilization was considered positive (green) if one of the associated pathways was confirmed by gapseq. If none of the associated pathways was found pathway utilization was considered negative (grey).

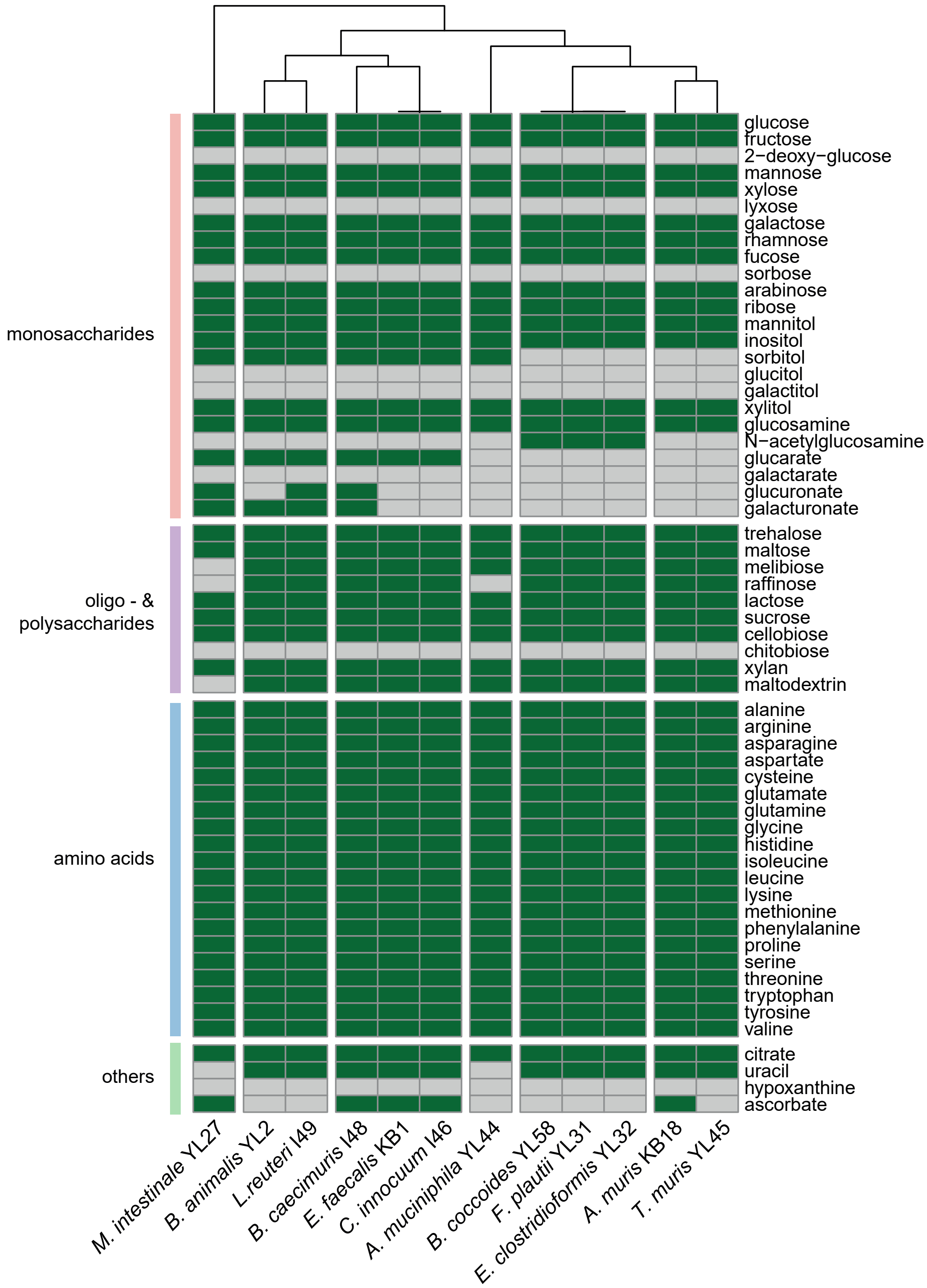

Fig. S11. Genome-informed potential for substrate transport. Different groups of substrate specific transporters were specified and gapseq output was mined for the corresponding transporter sequence IDs (SI data table 2). Positive hits are marked in green, negative hits in grey.

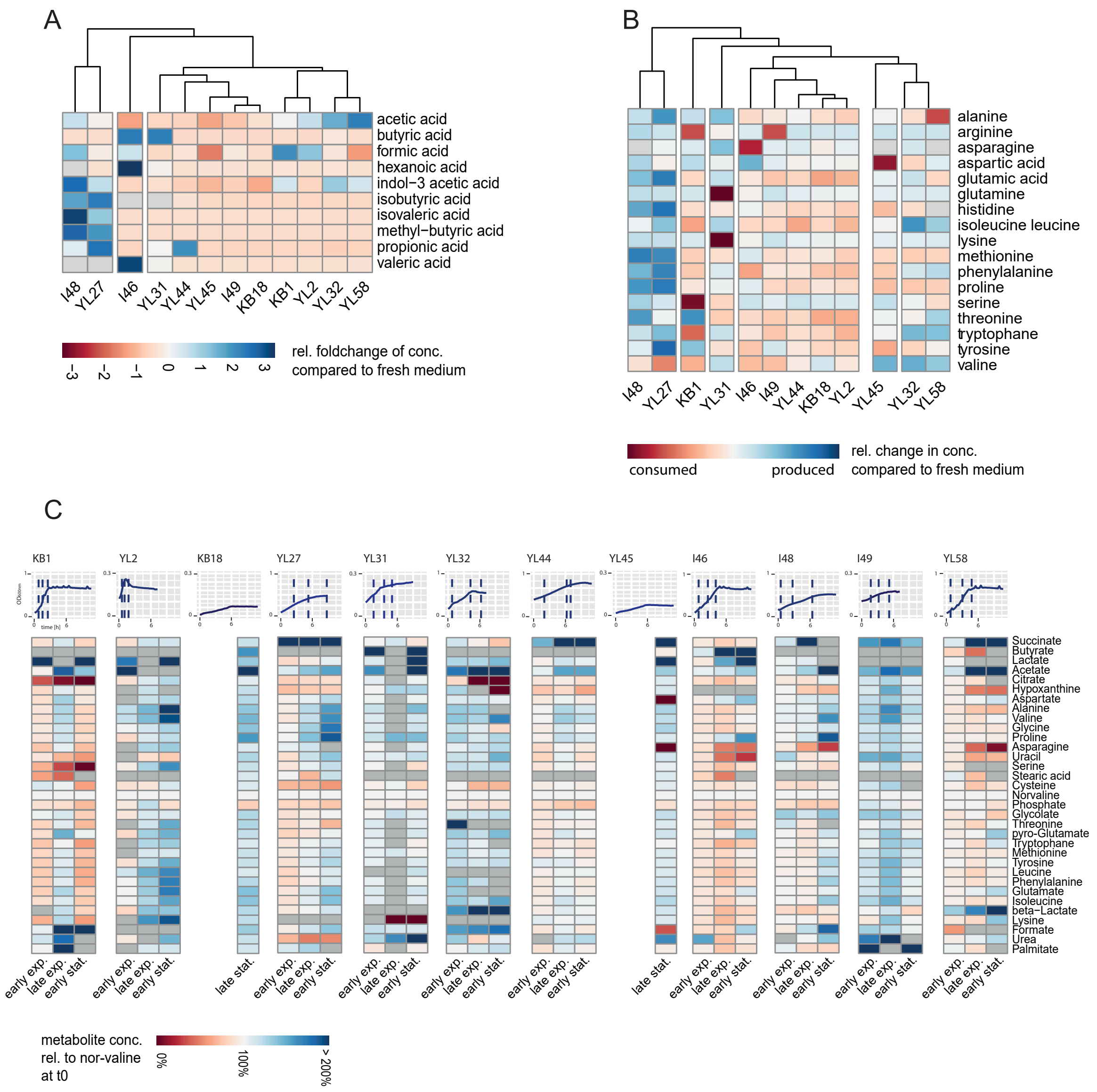

Fig. S 12. Metabolic profiling of SM. (**A**) SCFA profiles as determined by targeted metabolomics analysis of SM (late stationary phase, methods). SCFA consumption is indicated in red, while production is indicated in blue. (**B**) Amino acid profiles as determined by untargeted metabolomics analysis of SM (late stationary phase). Amino acids were annotated by comparing the exact mass and MS2 fragmentation patterns of the measured features to the records in HMBD (methods). Consumption of a specific amino acid from fresh medium is indicated in red, while production in indicated in blue. (**C**) Strain-specific metabolite profiles in different growth phases (early exponential, late exponential and early stationary phase) by GC-MS. Bacterial supernatants were sampled while simultaneously monitoring the individual growth stages by measuring OD at 600nm. Using an internal norvaline standard it was determined which metabolite levels increase (blue) and decrease (red) over time.

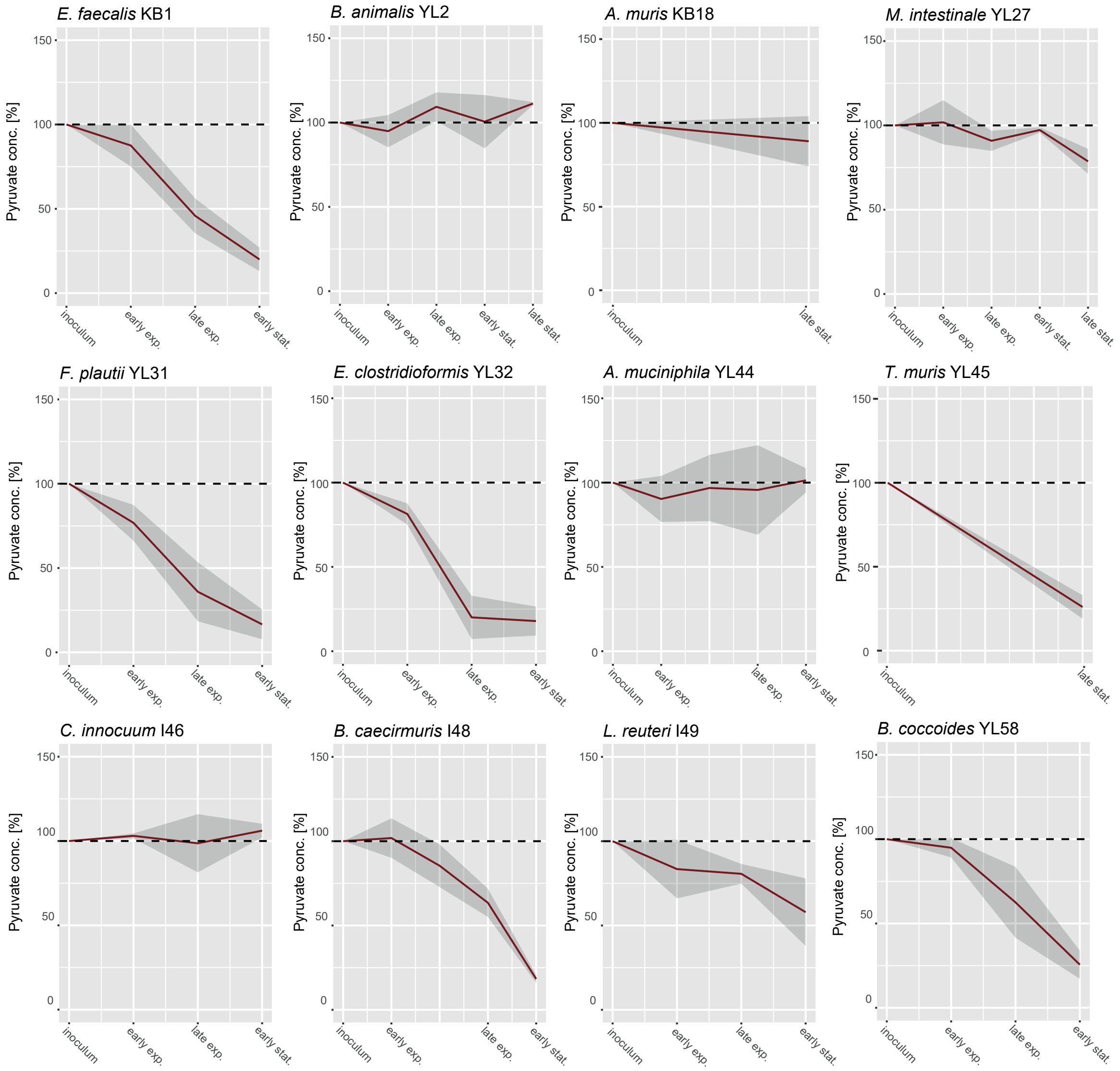

Fig. S13. Dynamic probing of pyruvate levels. Bacterial supernatants sampled at different time points of monoculture growth were spiked with ^{13}C labeled sodium pyruvate by GC-MS. Mean pyruvate levels (red line) are shown relative to fresh AF medium in percent with the corresponding standard deviation shown in grey.

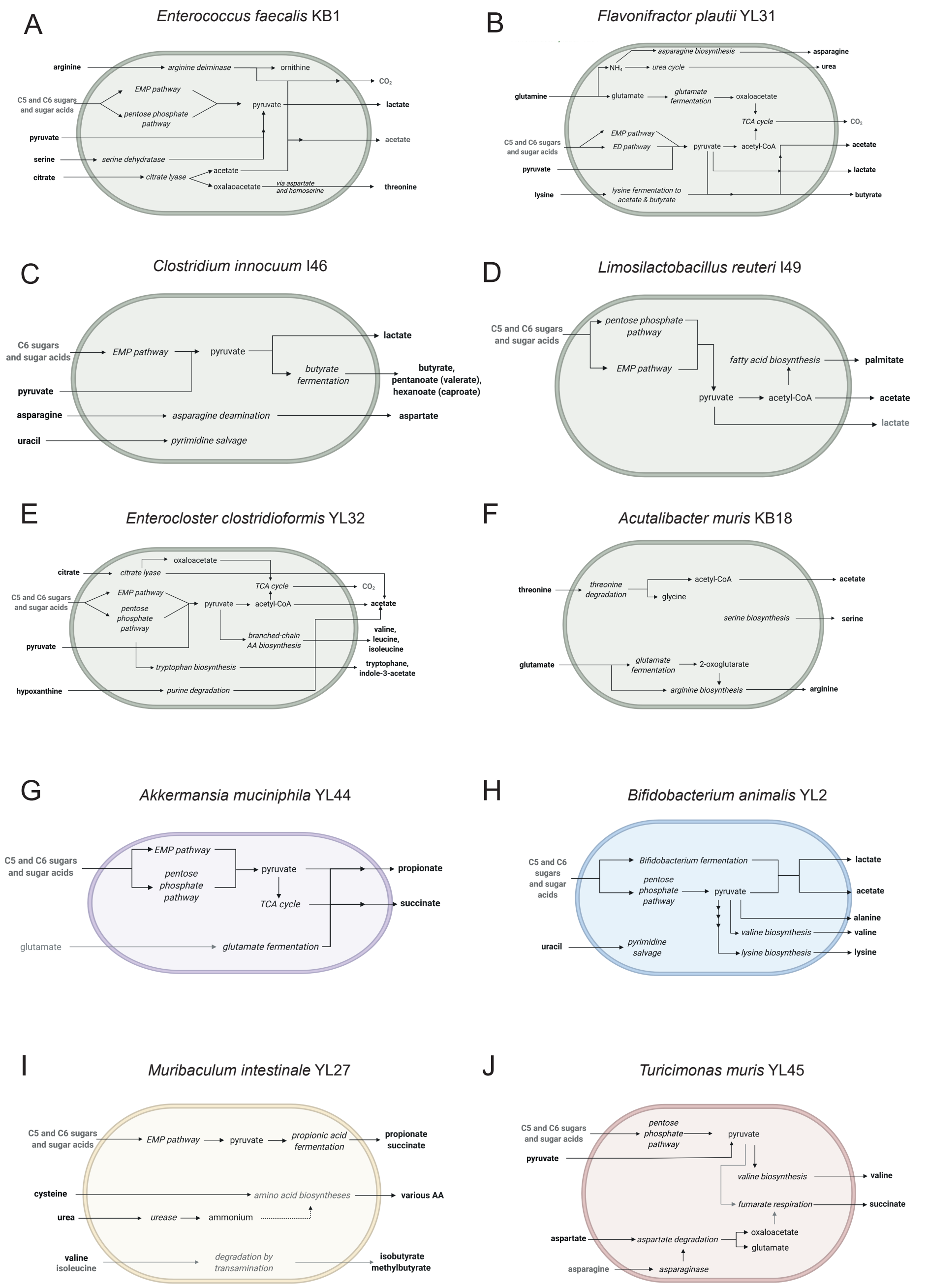

Fig. S14. Metabolic sekteches of the individual OMM^{12} community members. Combining metabolomics analyses with genome-based information on the presence of pathways enabled the generation of metabolic sketches of the individual OMM^{12} community members. Experimentally confirmed substrates, products, enzymes or pathways are shown in black. Hypothetical substrates, products, enzymes or pathways are shown in grey. Substrates, intermediate products or products are shown in roman, while enzymes or pathways are shown in italic font. Extracellular substrates or products are shown in bold. Additional information on the individual gapseq generated metabolic models can be found in SI data table 2.

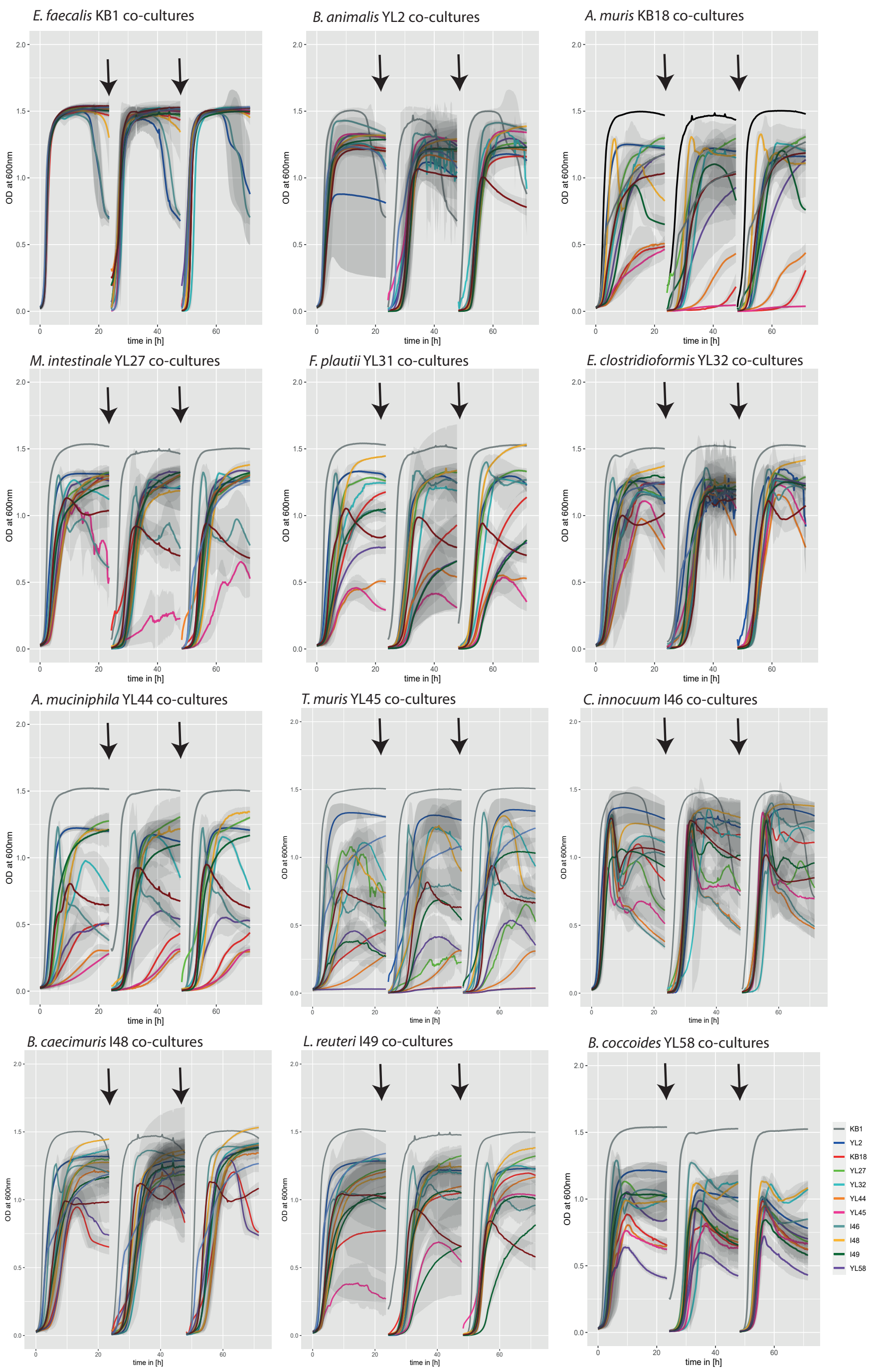

Fig. S15. Growth curves of all OMM^{12} bacteria in co-culture with all respective other strains. Growth of all co-cultures was monitored over 20 hours at OD 600nm. The corresponding monoculture is shown in black, while all respective co-cultures are shown in colored lines and standard deviations are shown in light grey. All cultures were diluted into fresh AF medium every 24 h for three days, dilutions are indicated with black arrows.

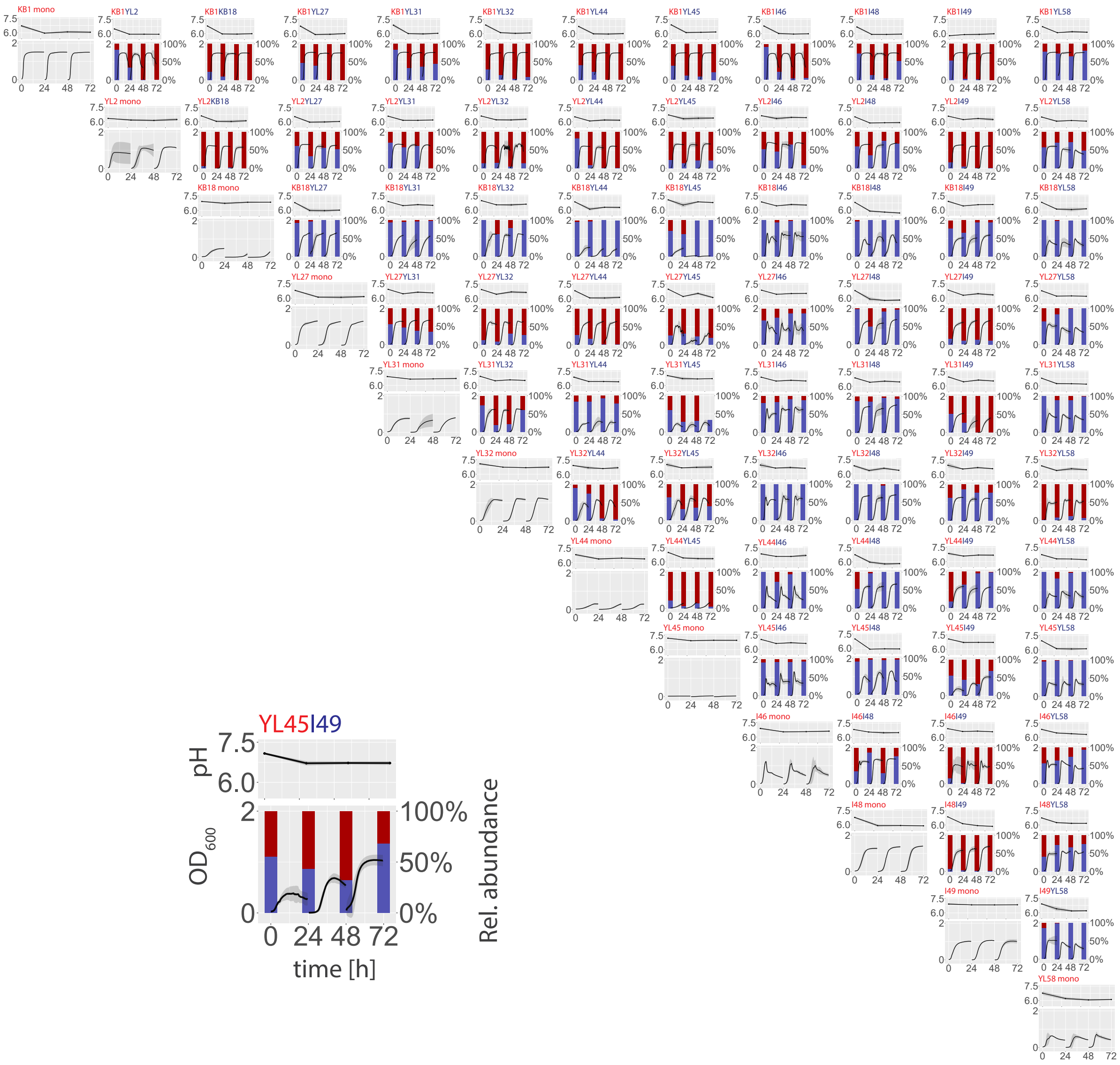

Fig. S16. Pairwise cultures of the OMM^{12} strains OMM^{12} pairwise strain combinations (12 monocultures, 66 co-cultures) were cultured in a 1:1 ratio in fresh AF medium over the course of 72 hours and growth, pH and relative abundance was monitored over time in three independent experiments. Growth at OD600nm and pH is shown as mean with the corresponding standard deviation (grey), relative abundance over time is shown exemplary for one of the three experiments. Examples of how growth curves develop with changing relative abundances is shown for the co-culture of *T. muris* YL45 and *L. reuteri* I49. Starting with a OD600nm ratio of approximately 1:1, final mean OD600nm values after the first two turnovers are low, corresponding to YL45 dominating the co-culture. After 48h, *L. reuteri* I49 resumes growth and final OD values increase. With *L. reuteri* I49 dominating the community in the end, the growth curve as well resembles *L. reuteri* I49 monoculture growth. Similarly, pH values reflect changes in co-culture structure.

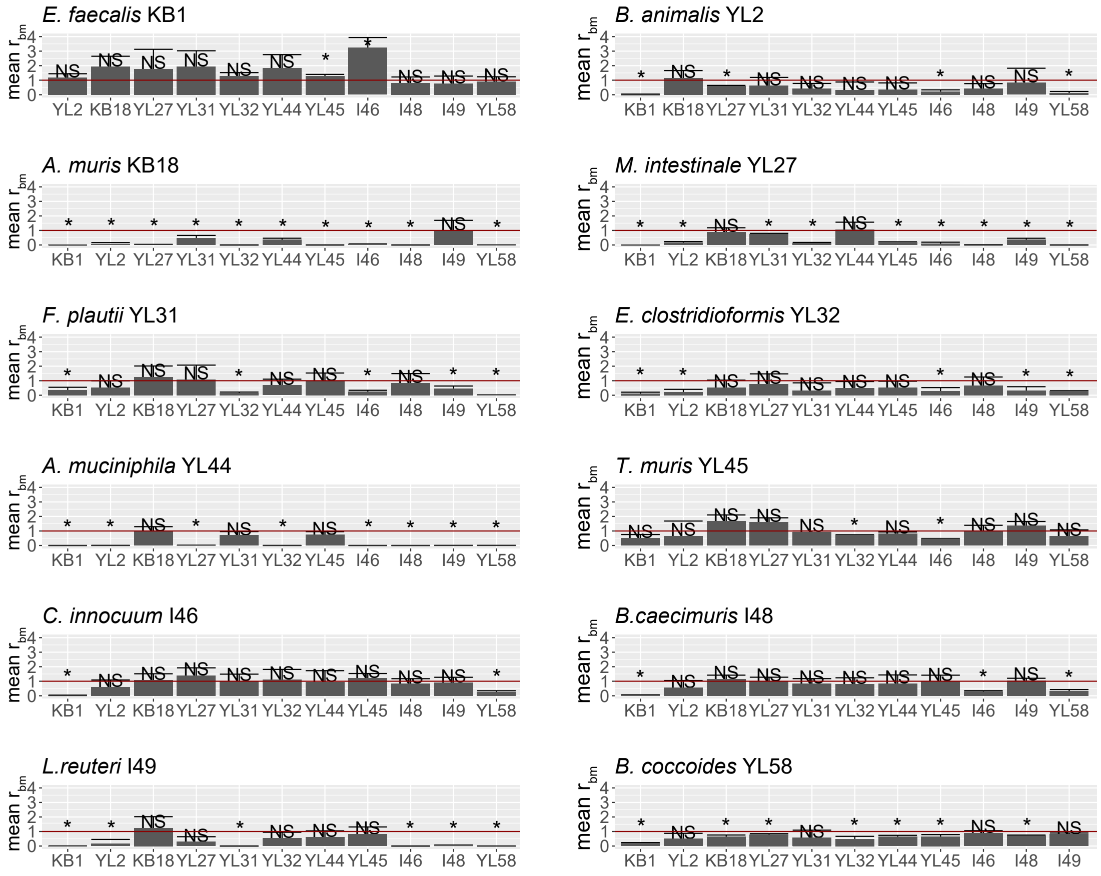

Fig. S17. Significance analysis of mean r_{bm} in all co-cultures. Mean absolute abundance ratios r_{bm} are shown as barplot with the corresponding standard deviation. The reference value (equal to one) is shown as red horizontal lines. A t-test was performed to determine significant increase or decrease in r_{bm} in the individual co-cultures relative to monoculture (r_{bm} =1). Significantly changed absolute abundance ratios r_{bm} ($p < 0.05$) are indicated by (*), non-significantly changed absolute abundance ratios r_{bm} are indicated by (NS).

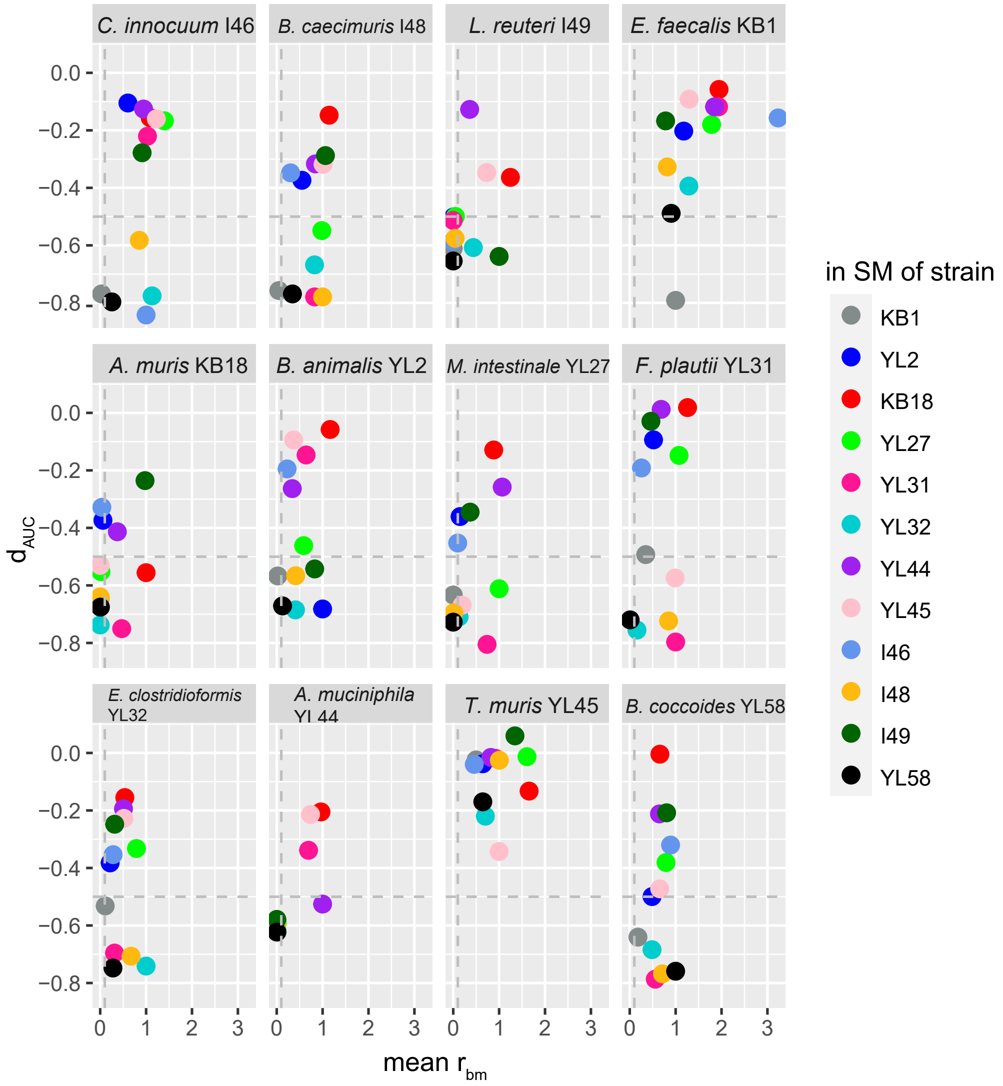

Fig. S18. Relationship between strain specific influence by SM and in co-culture. Comparing the influence of a strain on others in co-culture growth (r_{bm}) with the degree to which other strains were inhibited by its SM (d_{AUC}). Most co-cultures that resulted in the extinction of strain i, while strain j was not or positively affected ($r_{i,bm} \approx 0$, while $r_{j,bm} \geq 1$) also showed high inhibition values of strain i in the SM of strain j ($d_{i,AUC} \leq -0.5$). This suggests, that in most cases a strongly negative co-culture outcome for strain i corresponds to a strong inhibition of strain i by strain j due to specific waste or end products or substrate overlap.

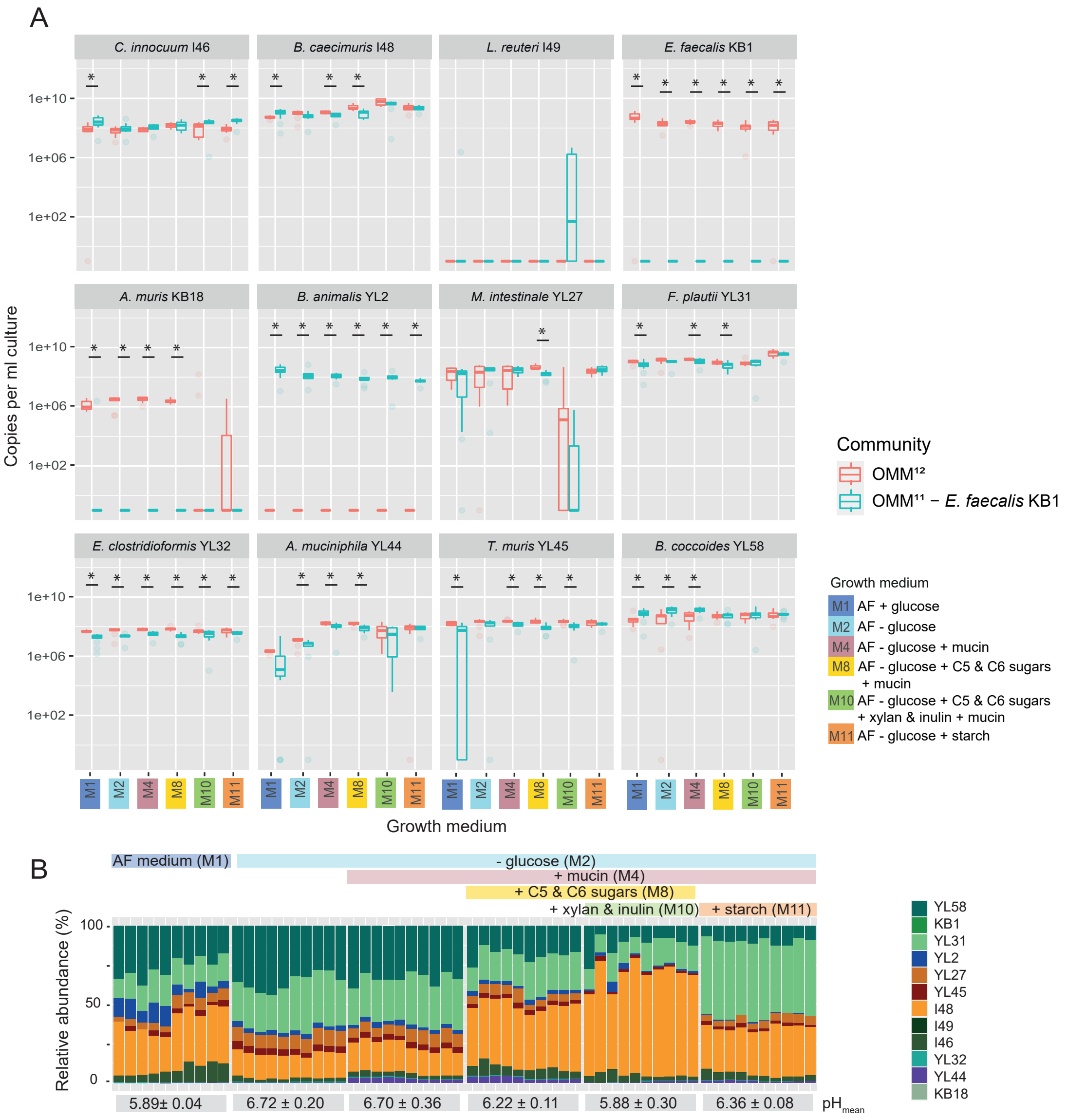

Fig. S19. Full OMM^{12} consortium and *E. faecalis* KB1 dropout community in different growth media. Absolute abundance of strains after ten days of serial dilution in batch culture in different growth media was determined by qPCR as normalized 16S rRNA copies per ml culture. Mean absolute abundances are shown with the corresponding SD for all individual strains. Strain *L. reuteri* I49 was below detection limit in the majority of communities in all replicates. Using a t-test, absolute abundances were compared between the full community cultures (N=10) and a dropout community lacking *E. faecalis* KB1 (N=10) for the individual growth conditions, p values < 0.05 are marked with * (A). Relative abundance of a OMM^{11}-*E. faecalis* KB1 dropout community in different growth media is shown for ten replicates each. Using a serial passaging batch culture setup, the community composition was analyzed after ten days of serial dilutions by comparing the relative strain abundances of ten replicates.The mean pH of all culture supernatants at day ten is shown with the corresponding SD (B).

A

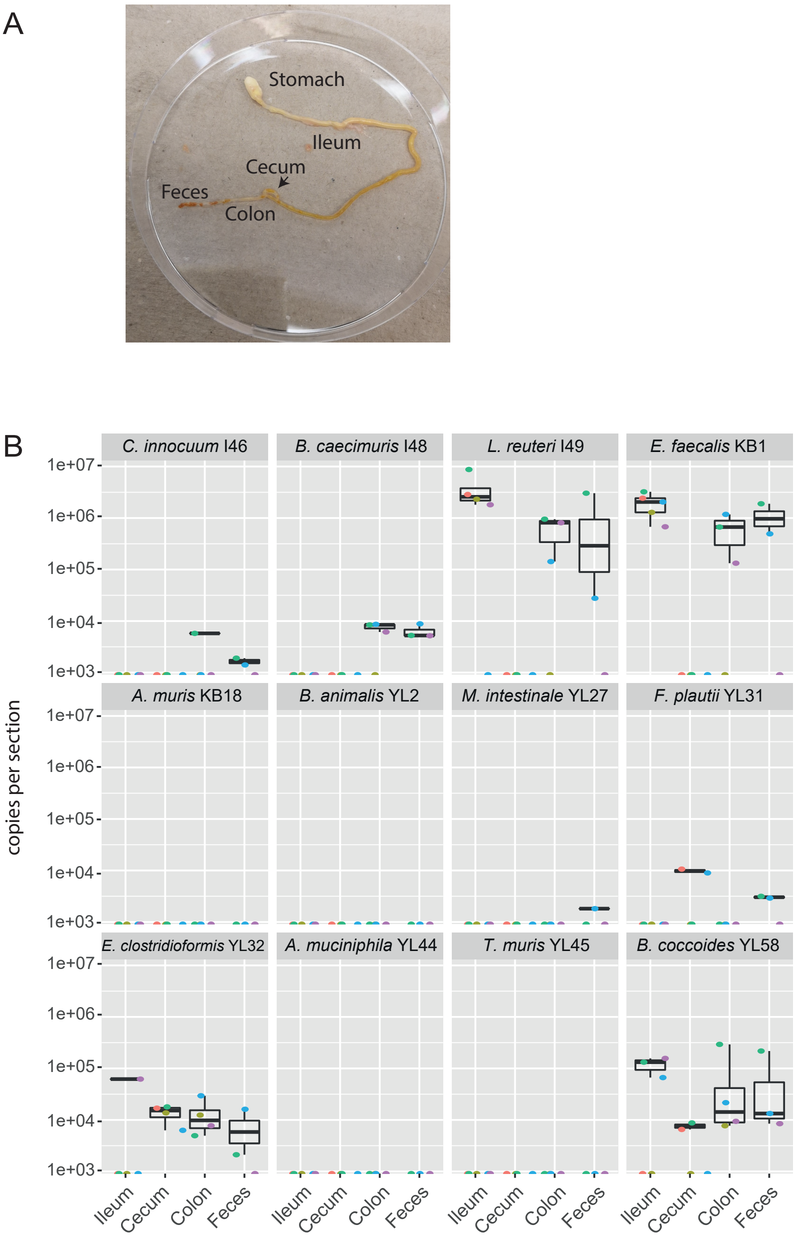

Fig. S 20. OMM^{12} community composition in the gut of infant mice. 7 day old infant mice were sacrificed and intestinal content from different gut regions was sampled. (A). Intestinal tract of mice with stomach and ileum (white), cecum and colon. (B) Absolute abundance of strains was determined by qPCR as normalized 16S rRNA copies per gut region section.

Supplemental Tables

Glucose
Fructose
Mannose
Galactose
Trehalose
Formate
Acetate
Propionate
Butyrate
Pyruvate
Lactate
Glycolate
Alanine
Glycine
β-Lactate
Urea
Valine
Norvaline
Leucine
Isoleucine
Succinate
Uracil
Proline
Phosphate
pyro-Glutamate
Methionine
Serine
Threonine
Phenylalanine
Aspartate
Cysteine
Hypoxanthine
Glutamate
Palmitate
Asparagine
Lysine
Stearat
Histidine
Citrate
Tyrosine
Tryptophan

Table S1. Analysis of metabolites in AF medium determined by GC-MS Using an internal norvaline standard, the listed metabolites were detected in AF medium. The list of compounds is non-comprehensive.

	mean GR [h^{-1}]	sd GR
E. faecalis KB1	2.40	0.11
B. animalis YL2	1.74	0.06
A. muris KB18	0.94	0.03
M. intestinale YL27	1.28	0.04
F. plautii YL31	1.13	0.02
E. clostridioformis YL32	1.33	0.04
A. muciniphila YL44	0.36	0.01
T. muris YL45	0.78	0.02
C. innocuum I46	1.83	0.08
B. caecimuris I48	1.26	0.05
L. reuteri I49	1.28	0.02
B. coccoides YL58	1.64	0.06

Table S2. Growth rates of the OMM^{12} strains in monoculture. Strain specific monoculture growth rates in AF medium were determined by time-resolved measurements of OD at 600nm and linear fitting of the exponential growth phase. Strains were grouped by growth rate (GR) into fast growing strains (shown in blue, GR > 1.5 h-1), strains with intermediate growth rate (shown in black, GR > 1 h-1) and slow growing strains (shown in red, GR < 1 h-1).

Supplemental Text A

Individual pH profiles as indicators for niche modification

The chemical composition of the individual SM is altered by substrate depletion and release of metabolic by- or waste products and change in pH. This change may lead to a different metabolic behavior of the strains while growing in SM. To quantify this, we determined the factor $\Delta\Delta$pH, which is the Euclidean distance between the ΔpH_{SM} that a strain shows after growth in fresh AF medium and the corresponding ΔpH_{DSM} after growth in each respective SM (**Fig. 1B and D**, **Fig. S6**, SI data table). This factor, $\Delta\Delta$pH, can reflect an altered metabolic behavior of a strain in a specific chemical environment in another strain's SM. For example, after growth of *B. coccoides* YL58 in fresh AF medium the strain specific ΔpH_{SM} was found as ΔpH_{SM} = -1.11 ($pH_{SM,\ YL58}$ = 5.89). After growth of *B. coccoides* YL58 in the SM of *L. reuteri* I49 ($pH_{SM,\ I49}$ = 6.90) the strain specific ΔpH_{DSM} was found as ΔpH_{DSM} = -0.58 (pH_{DSM} = 6.32), resulting in $\Delta\Delta$pH = 0.52. To identify cases, where products of a focal strain may lead to altered metabolic profiles of another strain, we correlated the $\Delta\Delta$pH values with the corresponding growth inhibition factor d_{AUC} (**Fig. S6**). We reasoned, that the metabolic effect (e.g. change in metabolic profile of a strain growing in a specific SM compared to fresh medium) is most pronounced if the overall growth is little affected (rel. change in AUC $d_{AUC} >$ -0.4), but the $\Delta\Delta$pH is large (Euclidean distance of pH profiles $\Delta\Delta pH > 0.5$). This was the case for *E. faecalis* KB1, *B. animalis* YL2, *M. intestinale* YL27, *B. cecimuris* I48 and *B. coccoides* YL58 in several different SM (**Fig. S6**, upper right quadrant). While these five strains were found to strongly acidify fresh culture medium during growth, they only weakly acidify the neutral SM of several other strains, including the SM of *A. muciniphila* YL44, *C. innocuum* I46 and *L. reuteri* I49. This suggests that the three latter strains alter their chemical environment in a way that somehow rewires metabolism of the others. The underlying changes may include the production of specific metabolic end products, e.g. SCFAs or depletion of favored growth substrates that require utilization of other metabolic pathways.

Supplemental Text B

Genes for the production of antibacterial compounds by *E. faecalis* KB1
The genome of *E. faecalis* KB1 (accession number CP022712.1) was screened for genes for the production of enterococcal bacteriocins (enterocins) using p-blast.

Query ID: **Enterocin L50A**
CP022712.1_2166 # 2333904 # 2334038 # -1 # ID=1_2166; partial=00; start_type=ATG; rbs_motif=GGAGG; rbs_spacer=5-10bp; gc_cont=0.281
Sequence ID: Query_66224 Length: 45
Range 1: 1 to 44
Score: 85.9 bits(211), Expect: 4e-26, Method: Compositional matrix adjust.,
Identities: 43/44 (98%), Positives: 44/44 (100%), Gaps: 0/44 (0%)
Query: MGAIAKLVAKFGWPIVKKYYKQIMQFIGEGWAINKIIEWIKKHI
Consence: MGAIAKLVAKFGWPIVKKYYKQIMQFIGEGWAINKII+WIKKHI
Subject: MGAIAKLVAKFGWPIVKKYYKQIMQFIGEGWAINKIIDWIKKHI

Query ID: **Enterocin L50B**
CP022712.1_2165 # 2333753 # 2333884 # -1 # ID=1_2165; partial=00; start_type=ATG; rbs_motif=AGGA/GGAG/GAGG; rbs_spacer=11-12bp; gc_cont=0.364
Sequence ID: Query_19555 Length: 44
Range 1: 1 to 43
Score: 83.2 bits (204), Expect: 4e-25, Method: Compositional matrix adjust.,
Identities: 40/43 (93%), Positives: 41/43 (95%), Gaps: 0/43 (0%)
Query: MGAIAKLVFKFGWPLIKKFYKQIMQFIGQGWTIDQIEKWLKRH
Consence: MGAIAKLV KFGWP IKKFYKQ+MQFIGQGWTIDQIEKWLKRH
Subject: MGAIAKLVAKFGWPFIKKFYKQVMQFIGQGWTIDQIEKWLKRH

Query ID: **Enterocin O16**
CP022712.1_26 # 23017 # 23529 # 1 # ID=1_26; partial=00; start_type=ATG; rbs_motif=AGxAGG/AGGxGG; rbs_spacer=5-10bp; gc_cont=0.366
Sequence ID: Query_4126 Length: 171
Range 1: 103 to 170
Score: 134 bits (338), Expect: 1e-43, Method: Compositional matrix adjust.,
Identities: 68/68 (100%), Positives: 68/68 (100%), Gaps: 0/68 (0%)
Query: LGSCVANKIKDEFFAMISISAIVKAAQKKAWKELAVTVLRFAKANGLKTNAI-IVAGQLAWAVQCGLSL
Consence: LGSCVANKIKDEFFAMISISAIVKAAQKKAWKELAVTVLRFAKANGLKTNAI-IVAGQLALWAVQCGLS
Subject: LGSCVANKIKDEFFAMISISAIVKAAQKKAWKELAVTVLRFAKANGLKTNAI-IVAGQLALWAVQCGLS

Query ID: **Enterocin 96**
CP022712.1_1870 # 2055051 # 2055275 # -1 # ID=1_1870; partial=00;start_type=ATG; rbs_motif=AGGAG; rbs_spacer=5-10bp; gc_cont=0.329
Sequence ID: Query_17682 Length: 75
Range 1: 27 to 74
Score: 102 bits (253), Expect: 4e-32, Method: Compositional matrix adjust.,
Identities: 48/48 (100%), Positives: 48/48 (100%), Gaps: 0/48 (0%)
Query: MSKRDCNLMKACCAGQAVTYAIHSLLNRLGGDSSDPAGCNDIVRKYCK

Consence: MSKRDCNLMKACCAGQAVTYAIHSLLNRLGGDSSDPAGCNDIVRKYCK
Subject: MSKRDCNLMKACCAGQAVTYAIHSLLNRLGGDSSDPAGCNDIVRKYCK
